# HOPS-dependent lysosomal fusion controls Rab19 availability for ciliogenesis in polarized epithelial cells

**DOI:** 10.1242/jcs.261047

**Published:** 2023-09-04

**Authors:** Huxley K. Hoffman, Rytis Prekeris

**Affiliations:** Department of Cell and Developmental Biology, University of Colorado Anschutz Medical Campus, Aurora, CO 80045, USA

**Keywords:** Cilia, Epithelial polarity, HOPS complex, Rab19, Renal epithelia, Lysosomal fusion

## Abstract

Primary cilia are sensory cellular organelles crucial for organ development and homeostasis. Ciliogenesis in polarized epithelial cells requires Rab19-mediated clearing of apical cortical actin to allow the cilium to grow from the apically docked basal body into the extracellular space. Loss of the lysosomal membrane-tethering homotypic fusion and protein sorting (HOPS) complex disrupts this actin clearing and ciliogenesis, but it remains unclear how the ciliary function of HOPS relates to its canonical function in regulating late endosome–lysosome fusion. Here, we show that disruption of HOPS-dependent lysosomal fusion indirectly impairs actin clearing and ciliogenesis by disrupting the targeting of Rab19 to the basal body, and that this effect is specific to polarized epithelial cells. We also find that Rab19 functions in endolysosomal cargo trafficking in addition to having its previously identified role in ciliogenesis. In summary, we show that inhibition of lysosomal fusion leads to the abnormal accumulation of Rab19 on late endosomes, thus depleting Rab19 from the basal body and thereby disrupting Rab19-mediated actin clearing and ciliogenesis in polarized epithelial cells.

## Teaser

**Summary:** Loss of HOPS-mediated lysosomal fusion indirectly blocks apical actin clearing and ciliogenesis in polarized epithelia by trapping Rab19 on late endosomes and depleting Rab19 from the basal body.

## INTRODUCTION

The primary cilium is a sensory organelle, found on most types of cells in vertebrates, which is required for many of the key cellular signaling pathways that coordinate organ development and homeostasis (Anvarian et al., 2019; Goetz and Anderson, 2010; Wheway et al., 2018). Mutations in genes involved in primary cilia formation and function produce genetic disorders termed ciliopathies, affecting multiple organ systems (Hildebrandt et al., 2011; Mitchison and Valente, 2017; Reiter and Leroux, 2017). Renal disease is a prominent feature of many ciliopathies, as defects in the primary cilia of the polarized epithelial cells that line the renal tubules lead to development of kidney cysts and loss of kidney function (Devlin and Sayer, 2019; McConnachie et al., 2021). For example, autosomal dominant polycystic kidney disease, the most common inherited cause of kidney failure, is caused by loss-of-function mutations in a ciliary receptor complex (McConnachie et al., 2021). This makes it important to understand the mechanisms underlying cilia formation in polarized epithelial cells.

Primary ciliogenesis involves extension of a microtubule-based axoneme from a basal body that develops from the mother centriole (Pedersen et al., 2008). The ciliogenesis process shows some mechanistic differences in polarized epithelial cells as compared to other cell types (Hoffman and Prekeris, 2022; Molla-Herman et al., 2010; Sorokin, 1968). In most cell types, the primary cilium initially develops within an intracellular ciliary vesicle that later fuses with the plasma membrane (PM) to expose the tip of the cilium to the extracellular space, but in polarized epithelial cells, it appears that the basal body first docks at the apical PM and then extends the cilium directly into the extracellular space. An important early step in this process is the formation of a clearing in the apical actin cortex (actin clearing) to allow the basal body docking, and protrusion and growth of the ciliary axoneme (Hoffman and Prekeris, 2022; Jewett et al., 2021).

Previous work from our laboratory (Jewett et al., 2021) identified the Rab19 GTPase and the homotypic fusion and protein sorting (HOPS) complex as being both required for this apical actin cortical-clearing step during ciliogenesis in renal polarized epithelial cells. Rab19 localizes to the site of actin clearing, and knockout (KO) of either Rab19 or the HOPS subunit Vps41 disrupts actin clearing and ciliogenesis. Another HOPS subunit, Vps39, has also been implicated in the regulation of ciliogenesis in renal cells (Iaconis et al., 2020), and all subunits of the HOPS complex were identified in a functional genomic screen for regulators of ciliary signaling (Breslow et al., 2018). The HOPS complex was also shown to be a Rab19 effector (Jewett et al., 2021).

Rab GTPases in general act as molecular switches that control intracellular membrane trafficking pathways (Homma et al., 2021; Stenmark, 2009), but specific roles of Rab19 outside of its requirement in ciliogenesis are largely unknown. Among the scarce clues to the overall functions of Rab19 is that the Rab19 ortholog in *Drosophila* localizes to the Golgi (Sinka et al., 2008). The HOPS complex, however, has a well-established role in lysosomal trafficking, where it serves as a membrane tethering complex for fusion of late endosomes (LEs), autophagosomes and AP-3 vesicles to lysosomes (hereafter referred to collectively as lysosomal fusion) (Balderhaar and Ungermann, 2013; Jiang et al., 2014)*.* Given that Rab19 showed little localization to LEs or lysosomes, it was proposed that Rab19 is not involved in lysosomal trafficking and that HOPS interacts with Rab19 to mediate actin clearing and ciliogenesis through a non-lysosomal pathway (Jewett et al., 2021). However, the mechanism by which HOPS regulates ciliogenesis, and whether Rab19 binding to HOPS is directly involved in apical actin clearance, remains unknown.

In this study, we investigated the role of the HOPS complex in actin cortical clearing and primary ciliogenesis. We found that pharmacological inhibition of lysosomal fusion produces similar defects in actin clearing and ciliogenesis to Vps41 KO, suggesting that the requirement for HOPS in ciliogenesis is a consequence of its requirement in lysosomal trafficking, as opposed to representing a non-lysosomal function of HOPS. We furthermore found that inhibiting lysosomal fusion impairs ciliogenesis not simply by blocking the degradation of ciliogenesis inhibitors, but rather by disrupting the regulation and localization of key ciliogenesis machinery (i.e. Rab19). The impact of impaired lysosomal fusion on ciliogenesis appears to be specific to polarized epithelial cells, as it was detected in two renal polarized epithelial cell lines (MDCK and mIMCD3) but not in a nonpolarized cell line (RPE1). Additionally, we discovered that Rab19 interacts not only with the late endosome and lysosome (LE/L)-associated HOPS complex but also with the related early endosome (EE)-associated class C core vacuole/endosome tethering (CORVET) complex, and we found that CORVET-containing EEs (in contrast to HOPS-containing LE/Ls) might be directly involved at the basal body in the process of ciliogenesis. We also found evidence for a previously uncharacterized role for Rab19 in endolysosomal trafficking. In summary, this study suggests that Rab19 has at least two distinct functions: (1) regulating lysosomal protein traffic, and (2) regulating apical actin clearance and cilia formation in polarized epithelial cells. In contrast, HOPS depletion appears to affect cilia formation indirectly, predominately by trapping Rab19 on enlarged LEs away from the apically localized basal body.

## RESULTS

### Disruption of lysosomal fusion impairs apical actin clearing and primary ciliogenesis

The HOPS complex regulates lysosomal cargo trafficking by acting as a membrane tether to mediate lysosomal fusion (Balderhaar and Ungermann, 2013; Jiang et al., 2014; Seals et al., 2000; Wartosch et al., 2015). Thus, it might be that HOPS affects ciliation by affecting the lysosomal degradation pathway, or alternatively it might be that HOPS has an additional non-lysosomal function in directly mediating traffic to and from the basal body. Consistent with the former possibility, it has been reported that autophagic degradation of several ciliation inhibitors is needed for ciliogenesis (Liu et al., 2021; Tang et al., 2013; Yamamoto and Mizushima, 2021; Yamamoto et al., 2021). Thus, we first assessed whether the requirement for the HOPS complex in actin cortical clearing and ciliogenesis was related to, or independent of, the role of HOPS in lysosomal fusion. To address this question, we tested whether pharmacological inhibition of lysosomal fusion would produce the same defects in actin clearing and ciliogenesis as Vps41 KO. Chloroquine (CQ) impairs lysosomal fusion (Mauthe et al., 2018; Mullock et al., 1998), making it a good candidate to pharmacologically mimic the lysosomal trafficking defect of Vps41 KO.

**Fig. 1.**
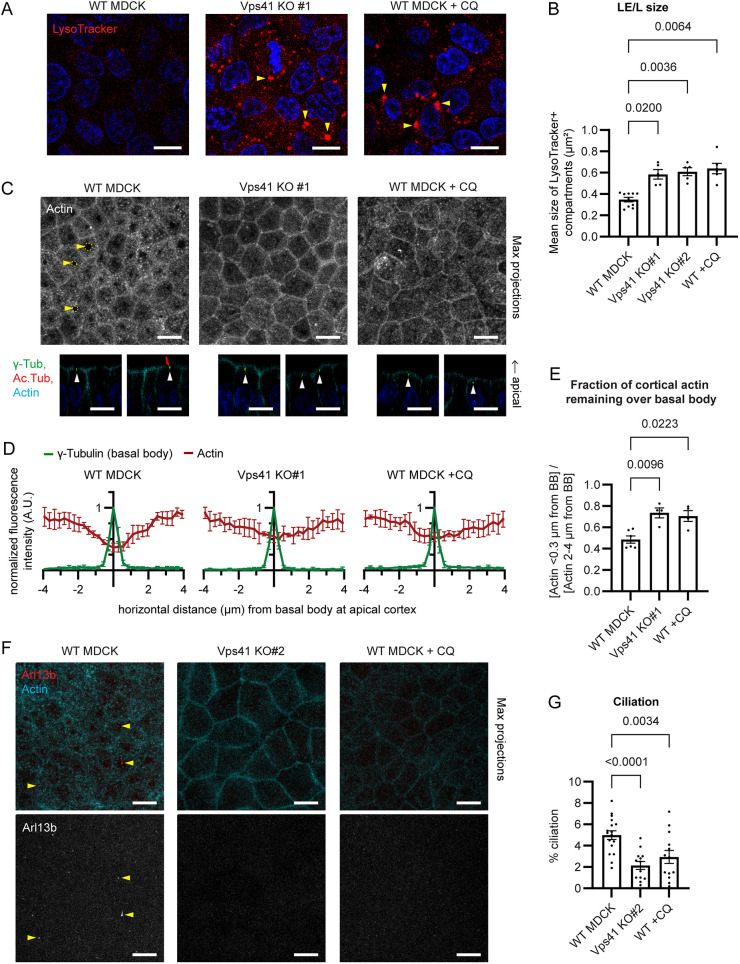
**Impaired lysosomal fusion disrupts actin cortical clearing and primary ciliogenesis.** (A) Parental control wild-type (WT), Vps41 KO or chloroquine-treated (+CQ; 10 µM for 2 days) MDCK cells, stained with LysoTracker to label late endosomes and lysosomes (LE/Ls; arrowheads). Both Vps41 KO and CQ-treated cells show enlarged LE/Ls (examples indicated with arrowheads). (B) Mean cross-sectional area of LysoTracker-stained LE/Ls, from experiments as in A. Each point represents the mean of technical replicates from one biological replicate. Outlier replicates excluded (ROUT=2%). Error bars are s.e.m. *P*-values calculated with a Brown–Forsythe and Welch ANOVA and Dunnett's T3 multiple comparisons test. (C) WT, Vps41 KO or CQ-treated (10 µM for 3 days) MDCK cells, stained with anti-γ-tubulin antibody (γ-Tub) to label basal bodies, anti-acetylated α-tubulin antibody (Ac. Tub) to label primary cilia and phalloidin to label actin. Upper panels, maximum intensity projection (MIP) of phalloidin; yellow arrowheads and circles indicate examples of actin cortical clearing. Lower panels, side views of merged channels; white arrowheads indicate basal bodies. Both Vps41 KO and CQ-treated cells show reduced clearing of apical cortical actin around the basal body. (D) Actin intensity profiles, from images as in lower panels of C (4–6 biological replicates with 10–15 technical replicates each). Error bars are s.d. (E) Ratio of cortical actin intensity directly above the basal body (within 0.3 μm laterally) to cortical actin intensity flanking the basal body (2–4 μm laterally), from the intensity profiles in D. Error bars are s.e.m. *P*-values calculated with a Brown–Forsythe and Welch ANOVA and Dunnett's T3 multiple comparisons test. (F) WT, Vps41 KO or CQ-treated (10 µM for 3 days) MDCK cells, stained with anti-Arl13b antibody to label primary cilia (arrowheads, examples of cilia) and phalloidin. Images are MIPs. Both Vps41 KO and CQ-treated cells show a lack of cilia. (G) Percentage of cells exhibiting an Arl13b-labeled primary cilium, from experiments as in F. Error bars are s.e.m. *P*-values calculated with a mixed-effects analysis with Geisser–Greenhouse correction and Šidák's multiple comparisons test. For all MDCK immunofluorescence experiments, polarized MDCK monolayers were grown on Transwell filters in complete medium. Blue in immunofluorescence images shows Hoechst 33342 staining of nuclei. Scale bars: 10 μm.

First, to confirm whether CQ had similar effects on the lysosomal pathway to Vps41 KO, we compared the morphology of acidic organelles, that is LE/Ls, in Vps41 KO Madin–Darby canine kidney (MDCK) cells to that in CQ-treated and untreated control wild-type (WT) MDCK cells. LysoTracker dye was used to label all acidic organelles, including LE/Ls. Both Vps41 KO and CQ treatment produced a dramatic increase in the size of LysoTracker-positive LE/Ls (Fig. 1A,B). These enlarged LysoTracker compartments likely represent stalled LEs, which have acidified enough for LysoTracker staining, but have failed to fuse to a mature lysosome to degrade their contents and therefore accumulate an abnormal volume of excess cargo. This result supports that CQ treatment mimics the lysosomal fusion defect of Vps41 KO.

We then used CQ as a tool to test whether inhibition of lysosomal fusion was sufficient to cause defects in apical actin clearing and ciliogenesis like those observed in Vps41 KO MDCK cells. The reduction in cortical actin above the basal body relative to flanking regions of the apical actin cortex (Fig. 1C–E; Fig. S1A), and the percentage of cells exhibiting a primary cilium after 3 days of culture as polarized monolayers (Fig. 1F,G; Fig. S1B), were compared between untreated control, CQ-treated, and Vps41 KO cells. Ciliation was assessed after 3 days because cells could withstand this duration of 10 µM CQ without major impacts to cell viability; the early time point accounts for the lower rate of ciliation in WT MDCK as compared to the prior study where ciliation was assessed after 8 days (Jewett et al., 2021). Actin clearing and ciliation were similarly impaired in both Vps41 KO and CQ-treated cells (Fig. 1C–G; Fig. S1), indicating that pharmacological inhibition of lysosomal fusion recapitulates the Vps41 KO defects in these processes. These findings suggest that the actin-clearing and ciliogenesis defects of Vps41 KO MDCK are likely due to the loss of the canonical HOPS complex function in lysosomal fusion, rather than reflecting a separate non-lysosomal function of the HOPS complex.

The clearing in the apical actin cortex around the base of the cilium, besides being important to allow for basal body docking and axoneme extension during ciliogenesis (Jewett et al., 2021), has also been shown to regulate the exclusion of non-ciliary apical membrane proteins such as gp135 (also known as PODXL) (Francis et al., 2011). Consistent with this, in CQ-treated cells as in Vps41 KO cells where actin clearing was defective, the clearing of gp135 was similarly lost (Fig. S2A). Although the clearing of basal-body-associated cortical actin is impaired in the majority of Vps41 KO and CQ-treated cells, a few cells in these conditions still exhibit normal actin clearing (Fig. S2B), and the few cilia that were observed in Vps41 KO and CQ-treated cells were associated with actin clearings (Fig. S2C). Thus, the occasional cilia found in Vps41 KO or CQ-treated cells represent the occasional cells in which actin clearing was successful, not cells where ciliogenesis proceeded without actin clearing, further demonstrating that ciliogenesis is tightly coupled to actin cortical remodeling. Although cilia were typically not found without actin clearings, actin clearings without detectable ciliary axonemes were frequently observed (Fig. 1C; Fig. S2D), supporting the model in which actin cortical clearing is required at an early stage of ciliogenesis prior to axoneme extension (Jewett et al., 2021) and thus the actin-clearing defect of Vps41 KO or CQ-treated cells is presumably a cause, rather than a consequence, of the ciliation defect.

### The ciliation defect of Vps41 KO MDCK cells is not due to impaired degradation of MYH9, OFD1 or CP110

**Fig. 2.**
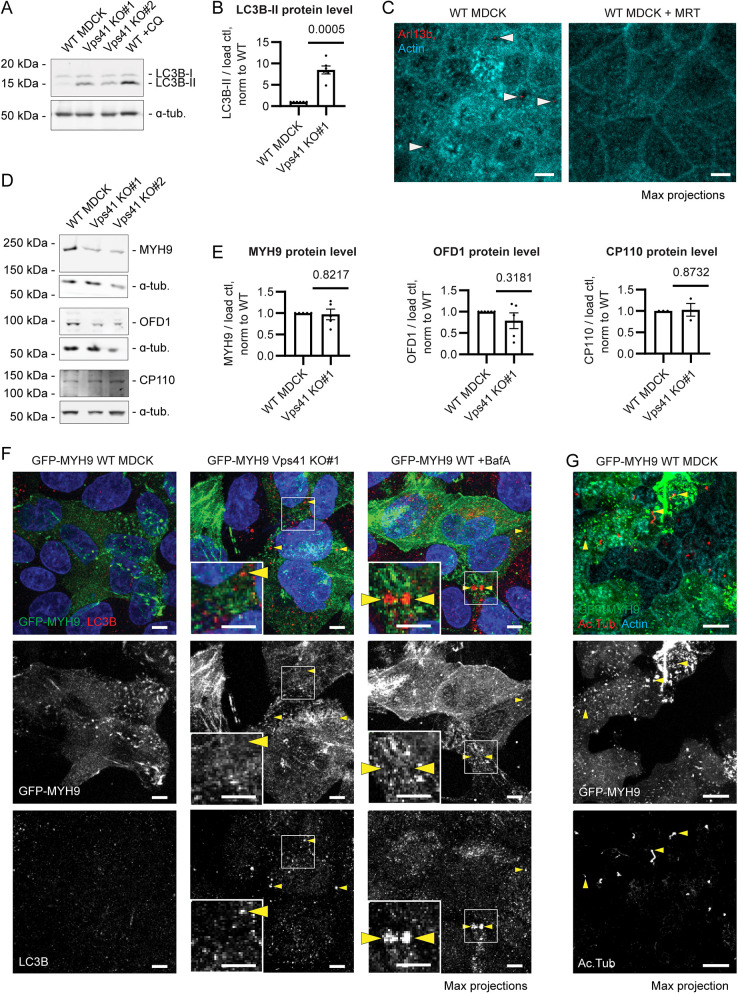
**The ciliation defect in Vps41 KO MDCK cells correlates with impaired autophagy but is not due to lack of degradation of MYH9, OFD1 or CP110.** (A) Western blot for autophagosome marker LC3B-II in WT, Vps41 KO and CQ-treated (10 μM for 2 days) MDCK cells. LC3B-II protein levels are elevated in Vps41 KO and CQ-treated cells, reflecting defects in autophagy. (B) Quantification of relative LC3B-II protein level for Vps41 KO #1 compared to WT MDCK, from western blots as shown in A. Error bars are s.e.m. *P*-values calculated with a one-sample *t*-test. (C) WT or MRT68921-treated (+MRT; 600 nM for 3 days) MDCK cells, stained with anti-Arl13b antibody to label primary cilia (arrowheads, examples of cilia) and phalloidin. Images are MIPs. The autophagy inhibitor MRT68921 blocked actin cortical clearing and ciliogenesis. (D) Western blots for MYH9, OFD1 and CP110. Levels of these proteins were not significantly altered in Vps41 KO as compared to WT MDCK. (E) Quantification of MYH9, OFD1 and CP110 relative protein levels for Vps41 KO #1 as compared to WT MDCK, from blots as shown in D. Error bars are s.e.m. *P*-values calculated with a one-sample *t*-test. (F) WT, Vps41 KO and BafA-treated (100 nM for 16 h) MDCK cells stably overexpressing GFP–MYH9 and stained with anti-LC3B antibody. MYH9 was not accumulated in autophagosomes in either Vps41 KO or +BafA conditions (arrowheads, examples of autophagosomes). (G) WT MDCK cells stably overexpressing GFP-MYH9 and stained with acetylated ɑ-tubulin antibody and phalloidin. MYH9 overexpression did not block actin clearing and ciliogenesis (arrowheads, examples of cilia). For all MDCK immunofluorescence experiments, polarized MDCK monolayers were grown on Transwell filters in complete medium. Blue in immunofluorescence images shows Hoechst 33342 staining of nuclei. Scale bars: 10 μm (C,G); 5 μm (F).

Previous research has found that autophagy promotes ciliogenesis by selectively degrading certain negative regulators of ciliogenesis, including MYH9 (Yamamoto et al., 2021), OFD1 (Tang et al., 2013) and CP110 (Liu et al., 2021). Autophagic degradation requires HOPS-mediated autophagosome–lysosome fusion (Jiang et al., 2014), and is therefore expected to be impaired by Vps41 KO. We confirmed that Vps41 KO MDCK cells, like CQ-treated cells, showed elevated levels of the autophagosome marker LC3B-II (lipidated form; LC3B is also known as MAP1LC3B) reflecting defects in autophagy (Fig. 2A,B). Furthermore, treatment with the autophagy inhibitor MRT68921, which blocks autophagy initiation by inhibiting ULK1 (Petherick et al., 2015), caused similar defects in actin clearing and ciliogenesis to those seen in Vps41 KO or CQ-treated cells (Fig. 2C), consistent with the idea that these defects might be due to the disruption of autophagy. We therefore wondered whether the ciliogenesis defects observed in Vps41 KO were due to impaired degradation of MYH9, OFD1 or CP110. MYH9 has been reported to inhibit ciliogenesis by stabilizing actin (Rao et al., 2014; Yamamoto et al., 2021), making it particularly likely that a lack of degradation of MYH9 could account for the actin-cortical-clearing defect. If MYH9 were an autophagy cargo and its degradation were blocked by Vps41 KO, then undegraded MYH9 would be expected to accumulate in autophagosomes, leading to an overall increase in MYH9 protein. However, we did not find any increase in the MYH9 protein level in Vps41 KO MDCK cells (Fig. 2D,E), nor did we see accumulation of GFP–MYH9 in autophagosomes either in Vps41 KO MDCK or in WT MDCK cells treated with the autophagy inhibitor Bafilomycin A1 (BafA) (Fig. 2F), suggesting that MYH9 is not an autophagy cargo in MDCK cells under the conditions of these experiments. We also examined the effects of GFP–MYH9 overexpression in WT MDCK cells and found that elevated levels of MYH9 did not block actin clearing or ciliation in these cells (Fig. 2G). Thus, contrary to previous reports in RPE1 and mIMCD3 cells (Rao et al., 2014), and MEFs (Yamamoto et al., 2021), MYH9 does not appear to be a ciliogenesis inhibitor in MDCK cells. Vps41 KO also showed no significant effect on OFD1 or CP110 protein levels (Fig. 2D,E). Our data therefore do not support the hypothesis that the actin-clearing and ciliogenesis defects of Vps41 KO cells are due to impaired autophagy of MYH9, OFD1 or CP110.

### Disruption of lysosomal fusion impairs ciliogenesis by retaining Rab19 on enlarged LEs and sequestering it away from the basal body

**Fig. 3.**
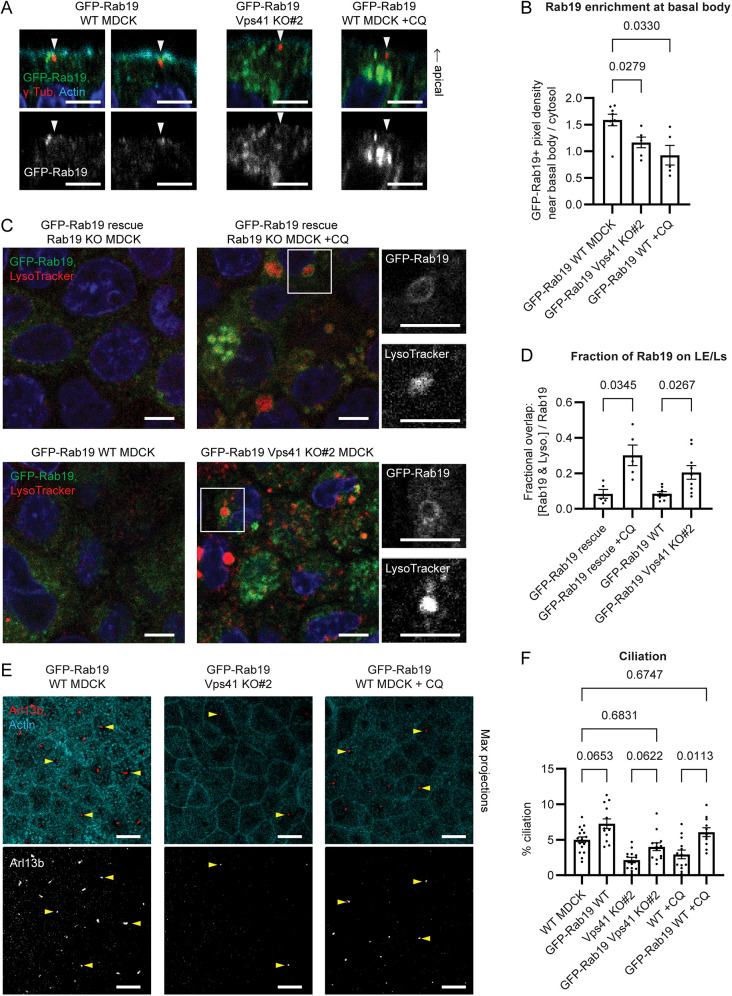
**Disruption of lysosomal fusion impairs ciliogenesis by relocalizing Rab19 to enlarged LEs away from the basal body.** (A) MDCK cells expressing GFP–Rab19 on a WT background with or without CQ treatment (10 μM for 3 days), or GFP–Rab19 on a Vsp41 KO background, stained with anti-γ-tubulin antibody and phalloidin. Basal body localization of Rab19 is decreased in Vps41 KO and CQ-treated cells. Images are side views. (B) Fold enrichment of GFP–Rab19 at the basal body, from experiments as in A. Error bars are s.e.m. *P*-values calculated with a Brown–Forsythe and Welch ANOVA and Dunnett's T3 multiple comparisons test. (C) MDCK cells expressing GFP–Rab19 on a Rab19 KO background with or without CQ treatment (10 μM for 2 days) (upper panels), or GFP–Rab19 on a WT or Vps41 KO background (lower panels), stained with LysoTracker. Rab19 shows little localization to LE/Ls under basal conditions, but accumulates on membranes of enlarged LE/Ls in CQ-treated or Vps41 KO cells. (D) Fractional overlap between GFP–Rab19 and LysoTracker, from experiments as in C. Error bars are s.e.m. *P*-values calculated with a Brown–Forsythe and Welch ANOVA and Dunnett's T3 multiple comparisons test. (E) GFP–Rab19-expressing WT, Vps41 KO and CQ-treated (10 μM for 3 days) MDCK cells, stained with Arl13b antibody and phalloidin. Images are MIPs. Arrowheads, examples of cilia. Compare to examples of non-GFP-Rab19-expressing parental cell lines in Fig. 1F. (F) Percentage of cells with an Arl13b-labeled primary cilium, from experiments as in E. Data for non-GFP–Rab19-expressing cell lines is the same as in Fig. 1G, repeated here for comparison to GFP–Rab19-expressing cell lines. Error bars are s.e.m. *P*-values calculated with a mixed-effects analysis with Geisser–Greenhouse correction and Šidák's multiple comparisons test. GFP–Rab19 overexpression enhanced ciliation in WT MDCK cells, partially rescued ciliation in Vps41 KO and fully rescued ciliation in CQ-treated cells. For all MDCK immunofluorescence experiments, polarized MDCK monolayers were grown on Transwell filters in complete medium. Blue in immunofluorescence images shows Hoechst 33342 staining of nuclei. Scale bars: 5 µm (A,C), 10 μm (E).

Given that Rab19 interacts with the HOPS complex and functions in actin cortical clearing and ciliogenesis (Jewett et al., 2021), we wondered whether the ciliogenesis defects caused by disrupted lysosomal trafficking were due to an effect on Rab19. We therefore examined whether Rab19 localization was altered in Vps41 KO or CQ-treated cells. As previously reported (Jewett et al., 2021), in WT MDCK cells, Rab19 vesicles were enriched around the basal body (Fig. 3A,B), and Rab19 showed little localization to LE/Ls (Fig. 3C,D; Fig. S3A). We also observed a substantial pool of Rab19 in the Golgi (Fig. S4A,B), consistent with the reported Golgi localization of Rab19 in *Drosophila* (Sinka et al., 2008). Although the Golgi localizes around the centrosome in many mammalian cell types (Masson and El Ghouzzi, 2022), in polarized MDCK cell monolayers, the Golgi was not detected at the actin-clearing site around the apically docked basal body, but rather closer to the nucleus (Fig. S4B), so the basal body localization of Rab19 appeared to be separate from its Golgi localization.

In Vps41 KO or CQ-treated MDCK cells, however, Rab19 localized strongly to the membranes of the enlarged LEs (Fig. 3C,D; Fig. S3A). This was accompanied by a reduction in basal body enrichment of Rab19 (Fig. 3A,B). These results indicate that impaired lysosomal fusion results in aberrant retention of Rab19 on LEs, thus, depleting the pool of Rab19 available to mediate actin clearing and ciliogenesis at the basal body. The portion of Rab19 localizing in the Golgi was also reduced in Vps41 KO or CQ-treated cells, although these conditions did not appear to grossly disrupt the structure of the Golgi itself (Fig. S4A,B), suggesting that the accumulation of Rab19 on LEs sequestered Rab19 away from its normal sites of action at both the basal body and the Golgi.

To assess whether depletion of Rab19 from the basal body was the mechanism for the actin-clearing and ciliogenesis defects in Vps41 KO and CQ-treated cells, we tested whether overexpression of Rab19 could rescue the defects. Indeed, GFP–Rab19 overexpression fully rescued actin clearing and ciliation in CQ-treated cells and partially rescued these phenotypes in Vps41 KO cells (Fig. 3E,F; Fig. S3B–D), and enhanced ciliation in control cells (Fig. 3E,F), supporting the hypothesis that mislocalization of Rab19 contributes to the actin-clearing and ciliogenesis defects produced by inhibition of lysosomal fusion (either by Vps41-KO or by CQ treatment). Importantly, Rab19 localization to the apical cortex was also lost in cells treated with the autophagy inhibitor MRT68921 (Fig. S3E), suggesting that depletion of Rab19 from the site of ciliogenesis could also be the mechanism for the actin-clearing and ciliation defects seen in MRT68921-treated cells (Fig. 2C).

### Relationship between lysosomal fusion and ciliogenesis is cell-type dependent

**Fig. 4.**
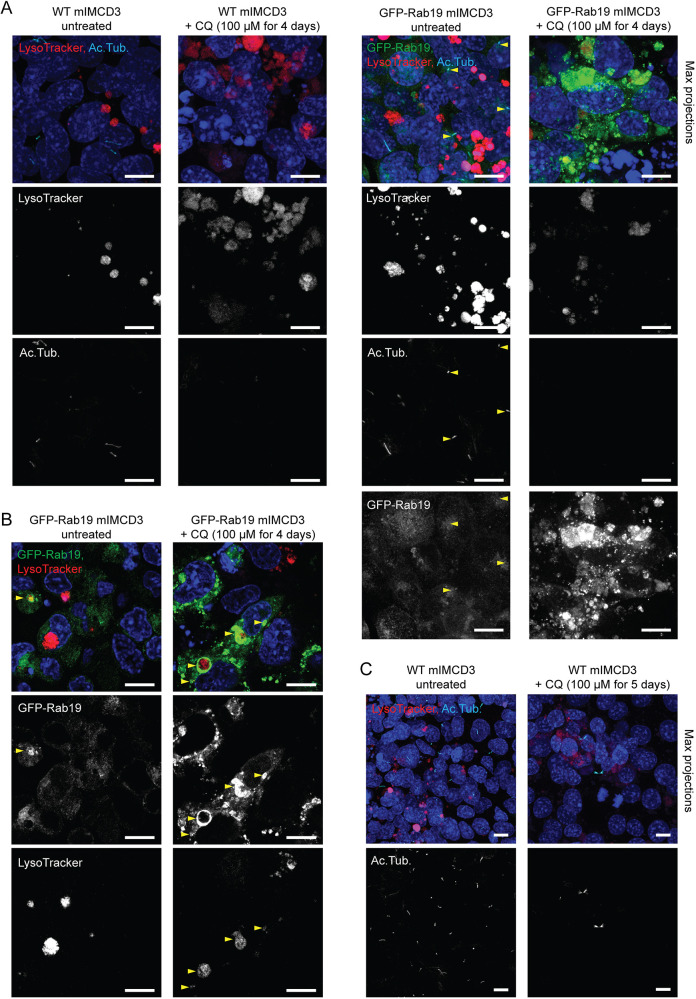
**Impaired lysosomal fusion increases Rab19 accumulation on LEs and disrupts primary ciliogenesis in polarized epithelial mIMCD3 cells.** (A) WT or GFP–Rab19-expressing mIMCD3 cells, with or without CQ (100 μM for 4 days), stained with LysoTracker and anti-acetylated α-tubulin antibody (Ac. Tub). Images are MIPs. CQ treatment produced more-irregularly-shaped LE/Ls with dimmer LysoTracker staining, increased Rab19 accumulation on LE/Ls and decreased Rab19 localization to the site of ciliogenesis (arrowheads, examples of Rab19 enrichment at the base of cilia), and blocked ciliogenesis. (B) GFP–Rab19-expressing mIMCD3 cells, with or without CQ (100 μM for 4 days), stained with LysoTracker. CQ treatment increased Rab19 localization to LE/Ls (examples indicated with arrowheads). (C) WT mIMCD3 cells, with or without CQ (100 μM for 5 days), stained with LysoTracker and anti-acetylated α-tubulin antibody. Images are MIPs. CQ treatment altered LE/L morphology and blocked ciliogenesis (arrowheads, examples of cilia; note that the prominent acetylated α-tubulin structures in the CQ-treated example are cytokinetic bridges, not cilia). All mIMCD3 monolayers were grown on Transwell filters in complete medium. Images are representative of at least seven fields of view per condition; the effects of CQ on LE/Ls and ciliation in mIMCD3 were also replicated in three separate experiments. Blue in immunofluorescence images shows Hoechst 33342 staining of nuclei. Scale bars: 10 μm.

To assess whether this mechanism was conserved in other polarized epithelial cells, we tested the effects of CQ in mouse inner medullary collecting duct-3 (mIMCD3) cells, which, like MDCK cells, are a renal epithelial cell line reported to use the extracellular ciliogenesis pathway (Molla-Herman et al., 2010). We found that mIMCD3 cells were less sensitive to CQ than MDCK cells, with concentrations up to 50 μM CQ showing little to no effect on Lysotracker compartments in mIMCD3 cells (data not shown). With 100 μM CQ, LysoTracker compartments were perturbed, indicating that 100 μM CQ was an effective dose to disrupt lysosomal fusion in mIMCD3, although the effects on LE/Ls appeared somewhat different in mIMCD3 than in MDCK cells; in mIMCD3 cells, which often exhibited large bright round LE/Ls in the untreated condition, CQ treatment produced more-irregularly-shaped LE/Ls with dimmer LysoTracker staining (Fig. 4A–C). 100 μM CQ treatment increased Rab19 accumulation on LE/Ls (Fig. 4A,B) and blocked ciliation in mIMCD3 cells (Fig. 4A,C). We also observed that ciliation in untreated mIMCD3 cells was enhanced by Rab19 overexpression, and that Rab19 was often enriched at the base of cilia (Fig. 4A), corroborating that Rab19 functions in ciliogenesis in this cell line. In short, inhibition of lysosomal fusion leads to the aberrant accumulation of Rab19 on LE/Ls and disruption of Rab19-mediated ciliogenesis in mIMCD3 cells, similar to the effects in MDCK, suggesting that this mechanism is conserved across polarized epithelial cell lines.

**Fig. 5.**
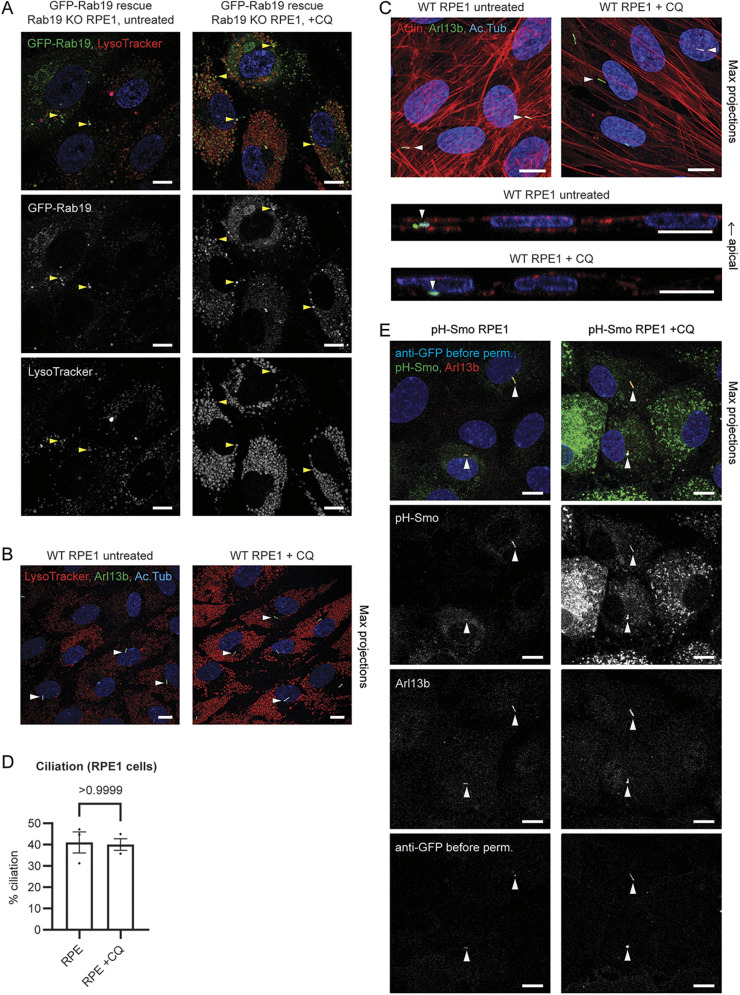
**Impaired lysosomal fusion increases Rab19 accumulation on LEs but does not block primary ciliogenesis in nonpolarized RPE1 cells.** (A) RPE1 cells expressing GFP–Rab19 on Rab19 KO background, with or without CQ (10 μM for 3 days), stained with LysoTracker; images are representative of at least six fields of view each. CQ treatment enlarged LE/Ls and increased Rab19 accumulation on LE/Ls (examples indicated with arrowheads). (B) WT RPE1 cells with or without CQ (10 μM for 3 days), stained with LysoTracker, anti-Arl13b antibody, and anti-acetylated α-tubulin antibody (Ac. Tub). Images are MIPs and are representative of two separate experiments with at least eight fields of view per condition per experiment. CQ treatment enlarged LE/Ls but did not block ciliogenesis (arrowheads, examples of cilia). (C) WT RPE1 cells with or without CQ (10 μM for 3 days), stained with anti-Arl13b antibody, anti-acetylated α-tubulin antibody and phalloidin; images are representative of at least 13 fields of view each. Upper panels are MIPs; lower panels are side views. Actin was mainly in stress fibers, with little cortical actin in comparison to MDCK cells (compare to Fig. 1C), and CQ treatment showed no major effects on actin organization or ciliation (arrowheads, examples of cilia). (D) Percentage of cells with an Arl13b-labeled primary cilium, from experiments as in B and C. Error bars are s.e.m. *P*-value calculated with a Wilcoxon test. Ciliation was not impaired by CQ treatment. (E) RPE1 cells expressing ciliary membrane marker pHluorin–Smoothened (pH-Smo), stained with anti-GFP antibody before permeabilization to label extracellularly exposed cilia (examples indicated with arrowheads), and with anti-Arl13b antibody after permeabilization to label all cilia; images are representative of at least 15 fields of view each. CQ treatment did not block extracellular exposure of cilia. All RPE1 samples were grown on coverslips and serum-starved for the final 2 days. Blue in immunofluorescence images shows Hoechst 33342 staining of nuclei. Scale bars: 10 μm.

We also tested the effects of CQ in human telomerase-immortalized retinal pigmented epithelial (hTERT-RPE1, herein abbreviated RPE1) cells, a non-polarized cell line that uses the intracellular ciliogenesis pathway (Molla-Herman et al., 2010). In RPE1 cells, as in MDCK, 10 μM CQ treatment increased the size and number of LysoTracker-positive LE/Ls, indicating that lysosomal fusion was impaired (Fig. 5A,B), and Rab19 localization to LE/Ls was also increased in CQ-treated RPE1 cells (Fig. 5A). Ciliation, however, was not impaired by CQ treatment in RPE1 cells (Fig. 5B–D), indicating that the relationship we had found between lysosomal fusion and ciliogenesis in polarized epithelial cells was not conserved in this non-polarized cell line.

Given that cilia in RPE1 cells form within an intracellular vesicle, which then fuses with the PM to expose the cilium to the extracellular space (Molla-Herman et al., 2010), we wondered whether actin cortical clearing in RPE1 cells might be needed to permit ciliary vesicle fusion to the PM, and whether this cilium exposure step might be blocked by CQ treatment although intracellular formation of the cilium was not. However, unlike the dense apical actin cortex in polarized MDCK monolayers, actin in RPE1 cells was found mainly in stress fibers and did not appear to be altered by CQ treatment (Fig. 5C). An IN/OUT assay (Kukic et al., 2016) using RPE1 cells expressing the ciliary membrane protein Smoothened with a pHluorin (a pH-sensitive GFP variant) tag and stained with anti-GFP antibody prior to permeabilization to label only extracellularly exposed cilia, showed that cilium exposure was not prevented by CQ treatment (Fig. 5E). Thus, although disruption of lysosomal fusion affects Rab19 localization in both polarized and non-polarized cells, the impact on actin clearing and ciliogenesis appears to be specific to polarized epithelial cells that use the extracellular ciliogenesis pathway.

### Rab19 functions in cargo transport to LEs

The observation that Rab19 accumulates on LEs in Vps41 KO or CQ-treated cells led us to investigate how Rab19 is involved in the endolysosomal pathway. Given that Rab19 showed relatively little localization to LE/Ls at steady state but accumulated on LEs when their fusion was blocked (Figs 3C,D, 4A,B and 5A; Fig. S3A), it is likely that under normal conditions, Rab19 is transiently recruited to LE membranes and then released from those membranes following lysosomal fusion. To investigate the function of Rab19 targeting to LE/Ls, we examined the LE/L phenotypes for Rab19 KO and for the Rab19 constitutively active (CA) mutant Q79L in MDCK cells. Neither Rab19 KO nor Rab19-CA showed any major effect on LE/L size in untreated cells (Fig. S4C,D). However, Rab19-CA expression exaggerated the CQ-induced enlargement of LE/Ls (Fig. S4C,D). A likely interpretation for this finding is that Rab19 mediates trafficking of certain cargoes to LE/Ls. Under conditions of normal lysosomal function, an altered rate of delivery of those cargoes might have a limited effect on LE/L size because the cargo is rapidly degraded in lysosomes, and thus it is only when lysosomal degradation is inhibited (e.g. by CQ treatment) that increased delivery of these cargoes results in increased swelling of the LE/Ls.

### Rab19 interacts with core subunits of both HOPS and CORVET complexes

To explore the significance of the Rab19–HOPS interaction, we set out to identify which subunit of the HOPS complex mediates its binding to Rab19. The HOPS complex contains two HOPS-specific subunits (Vps41 and Vps39) along with four core subunits (Vps11, Vps16, Vps18 and Vps33A), which are shared with the early endosomal CORVET complex (Balderhaar and Ungermann, 2013) (Fig. S5A).

As previously reported (Jewett et al., 2021), glutathione bead pulldown experiments with recombinantly produced GST–Rab19 confirmed that Rab19 in its GTP-bound state interacted with HOPS (as determined by immunoblotting for the endogenous Vps11 subunit) in WT MDCK cell lysates (Fig. S5B). We also detected the interaction of Rab19 with Vps11 in Vps41 KO MDCK lysates (Fig. S5B), suggesting that Rab19 binding to HOPS complex is not mediated by Vps41. To probe the dependence of Rab19–HOPS interaction on the other HOPS subunits, we used siRNA to deplete each of the HOPS subunits in HEK293T cells (Fig. S5C–E). Knockdown of any one of the HOPS/CORVET core subunits severely disrupted the entire HOPS complex, as indicated by decreased levels of Vps11 and Vps41 in cell lysates (Fig. S5C), so the roles of these core subunits in Rab19–HOPS binding could not be individually assessed. Knockdown of Vps39, in contrast, had only moderate effects on Vps11 protein levels, and did not impair the interaction of Rab19 with Vps11 (Fig. S5C,D). Thus, neither one of the HOPS-specific subunits, Vps41 or Vps39, appear to be required for the Rab19–HOPS interaction.

The HOPS complex binds Rab7 via both Vps41 and Vps39 (Brett et al., 2008; Bröcker et al., 2012). We wondered whether Rab19 might similarly interact with both Vps41 and Vps39, which could explain why knockdown of either Vps41 or Vps39 alone failed to disrupt the interaction. However, co-depletion of both Vps41 and Vps39 still did not reduce Rab19 binding to Vps11 (Fig. S5D,E). We further investigated whether Rab19 competes with Rab7 for binding to HOPS, by testing whether addition of purified recombinant Rab7A in the GST–Rab19 pulldown experiment would reduce Rab19–HOPS binding. Addition of Rab7 did not impair the Rab19–HOPS interaction (Fig. S5D,F), suggesting that Rab19 binds to HOPS at a different site than Rab7 does. All these results indicate that the binding determinants for Rab19 are distributed across multiple HOPS subunits beyond Vps41 and Vps39, suggesting that HOPS core subunits might mediate interaction with Rab19.

Given that HOPS and CORVET share the same core subunits, we then wondered whether Rab19 also interacts with the CORVET complex. To test this possibility, we performed GST–Rab19 pulldown assays using lysates of HEK293T cells overexpressing the CORVET-specific subunits HA–Vps3 or Vps8–GFP. HA–Vps3 and Vps8–GFP (presumably as part of CORVET complex) did bind to GST–Rab19 (Fig. S5G,H), supporting the hypothesis that Rab19 can also interact with CORVET. Collectively, these results suggest that Rab19 likely binds to HOPS/CORVET core subunits, and thus, likely interacts not only with HOPS but also with CORVET complex.

### EEs but not LE/Ls are found at the site of ciliogenesis

**Fig. 6.**
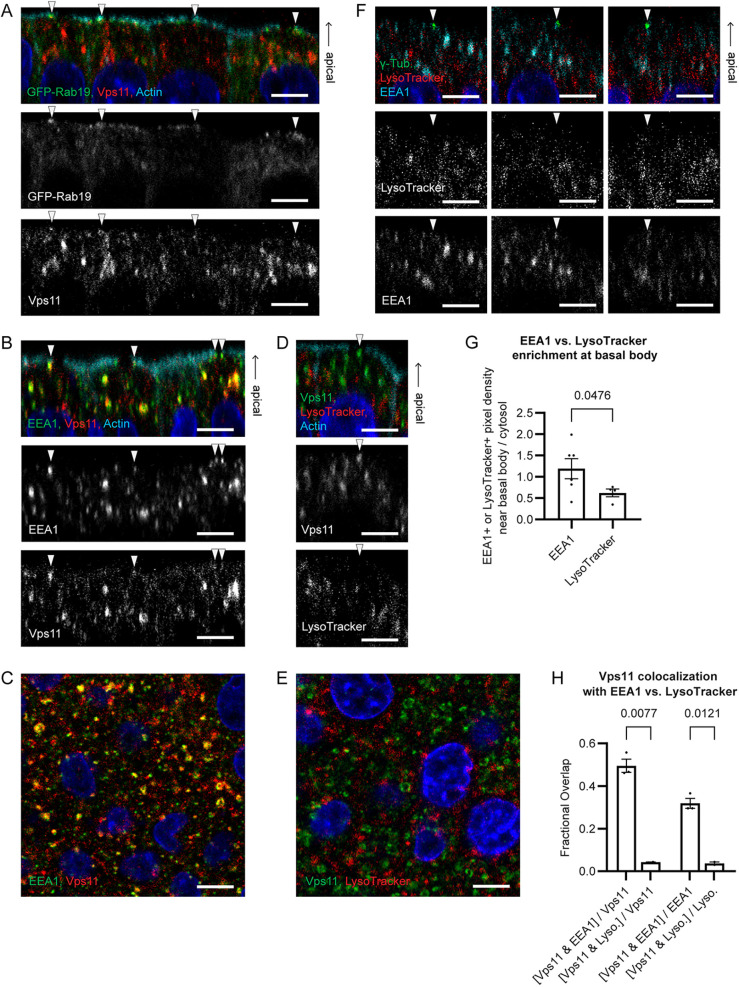
**CORVET-containing EEs are found at the site of ciliogenesis.** (A) GFP–Rab19-expressing WT MDCK cells stained with anti-Vps11 antibody and phalloidin. Images are side views and are representative of five fields of view. Vps11 overlaps with Rab19 at the periphery of the actin clearing (arrowheads). (B) WT MDCK cells stained with anti-Vps11 and -EEA1 antibodies and phalloidin. Images are side views. Vps11 at the actin-clearing site colocalizes with EEA1 (arrowheads). (C) WT MDCK cells stained with anti-Vps11 and -EEA1 antibodies. Vps11 colocalizes with EEA1. (D) WT MDCK cells stained with anti-Vps11 antibody, LysoTracker and phalloidin. Images are side views. Vps11 at the actin-clearing site (arrowhead) has little colocalization with LysoTracker. (E) WT MDCK cells stained with anti-Vps11 antibody and LysoTracker. Vps11 has little colocalization with LysoTracker. (F) WT MDCK cells stained with γ-tubulin and EEA1 antibodies and LysoTracker. Image are side views. EEA1 but not LysoTracker is observed at the basal body. (G) Fold enrichment of EEA1 or LysoTracker at the basal body, from experiments as in F. Error bars are s.e.m. *P*-values calculated with a Kolmogorov–Smirnov test. (H) Fractional overlap of Vps11 antibody with EEA1 antibody or with LysoTracker, from images as in C and E. Error bars are s.e.m. *P*-values calculated with a Brown–Forsythe and Welch ANOVA and Dunnett's T3 multiple comparisons test. For all MDCK immunofluorescence experiments, polarized MDCK monolayers were grown on Transwell filters in complete medium. Blue in immunofluorescence images shows Hoechst 33342 staining of nuclei. Scale bars: 10 μm.

If Rab19 binds to both HOPS and CORVET, we wondered whether either HOPS or CORVET are directly involved in the ciliogenesis function of Rab19. To test this question, we examined the subcellular localization of the HOPS/CORVET core by immunostaining for Vps11. Vps11 puncta were frequently observed at the periphery of the actin cortical clearing (Fig. 6A,B,D), where they sometimes overlapped with Rab19 (Fig. 6A), potentially supporting a direct role for HOPS and/or CORVET in ciliogenesis.

We were unable to directly assess the distinct localizations of HOPS and CORVET, because commercially available antibodies for the HOPS-specific and CORVET-specific subunits did not perform adequately for immunofluorescence in MDCK cells, and overexpression of tagged HOPS subunits disrupts the function of these complexes in ciliogenesis (unlike Rab19, for which overexpression of a tagged form enhances ciliation in WT cells and rescues ciliation in Rab19 KO, indicating that overexpressed Rab19 is functional in ciliogenesis) (Jewett et al., 2021). To distinguish whether the Vps11 at the site of ciliogenesis represented HOPS or CORVET, we therefore used immunostaining for early endosome antigen 1 (EEA1) as a marker for EEs (organelles containing CORVET), and LysoTracker as a marker for LE/Ls (organelles containing HOPS). EEA1, like Vps11, was often detected at the basal body and actin-clearing site (Fig. 6B,F,G), and Vps11 colocalized strongly with EEA1, both at this site and overall in the cells (Fig. 6B,C,H). In contrast, LysoTracker showed little localization to the ciliogenesis site (Fig. 6D,F,G) and little colocalization with Vps11 (Fig. 6D,E,H). Thus, it appears that in WT MDCK cells, the majority of Vps11, including the Vps11 present at the site of ciliogenesis, is part of CORVET complex on EEs, with relatively little Vps11 in HOPS complex on LE/Ls.

To probe whether EEs function in ciliogenesis or in subsequent trafficking to or from cilia, we also examined EEA1 localization with respect to Arl13b. EEA1-positive EEs were frequently found at sites of actin cortical clearing that did not show cilia, and also at the base of Arl13b-positive cilia (Fig. S6A), suggesting that these EEs might be involved both early in ciliogenesis and later in ciliary trafficking. We then tested whether Rab19 and its interaction with CORVET was responsible for targeting EEs to the ciliogenesis site. Rab19 KO MDCK cells also showed EEA1 at the basal body (Fig. S6B), indicating that Rab19 is not required for localizing EEs to this site. It might instead be that Rab19 transports some ciliogenesis factors to the EEs at the basal body to promote ciliogenesis. We also tested whether EEs at the site of ciliogenesis were perturbed by inhibition of lysosomal fusion. EEA1 was observed at basal bodies in Vps41 KO and CQ-treated as well as WT MDCK cells (Fig. S6C), indicating that inhibition of lysosomal fusion did not disrupt the targeting of EEs to the site of ciliogenesis, although it might well be that the contents of these EEs are dysregulated by accumulation of cargoes that would normally be transported to lysosomes and degraded.

**Fig. 7.**
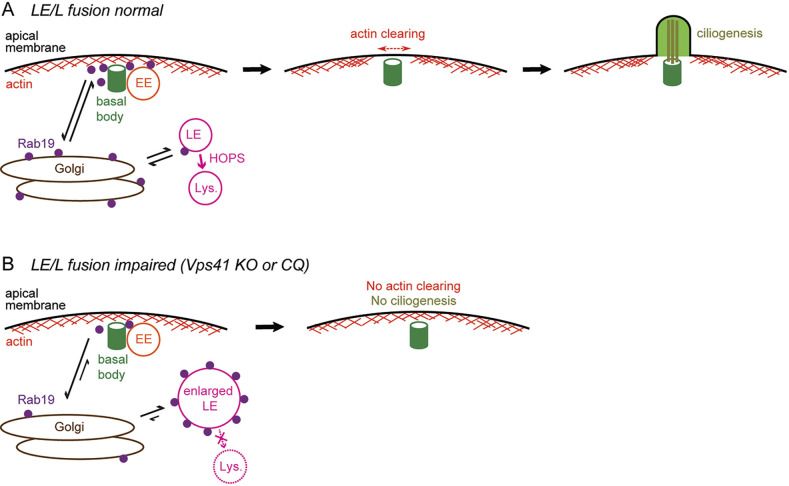
**Model of how HOPS-dependent lysosomal fusion regulates actin cortical clearing and ciliogenesis in polarized epithelial cells by controlling Rab19 availability at the basal body.** (A) Under normal conditions, a large portion of Rab19 is targeted to the basal body to drive apical actin cortical clearing and ciliogenesis, while a minor population of Rab19 is transiently recruited to LE membranes and then cycles off of these membranes following lysosomal fusion. EEs, but not LE/Ls, are enriched at the basal body. (B) When lysosomal fusion is impaired by Vps41 KO or CQ treatment, then Rab19 is not released from LEs, which become enlarged with undegraded cargo. Accumulation of Rab19 on LEs depletes the pool of Rab19 that can be recruited to the basal body, leading to defects in actin clearing and ciliogenesis.

These observations are consistent with the idea that Rab19–CORVET interaction might play a direct role in ciliogenesis, although further study will be needed to demonstrate that role. In contrast, we see little evidence for HOPS or LE/Ls at the site of ciliogenesis (although we cannot rule out the possibility that small LE/Ls with LysoTracker signal below the detection limit of this assay could be present there), suggesting that HOPS and its interaction with Rab19 are likely not directly involved in ciliogenesis. This is consistent with the model in which the ciliogenesis defects of Vps41 KO or CQ-treated cells are due to aberrant retention of Rab19 on LEs impeding recruitment of Rab19 to the site of ciliogenesis (Fig. 7).

## DISCUSSION

In this study, we set out to elucidate the role of the HOPS complex in apical actin cortical clearing and primary ciliogenesis. Knockout or mutation of HOPS subunits was recently shown to disrupt primary ciliogenesis and ciliary signaling (Breslow et al., 2018; Iaconis et al., 2020; Jewett et al., 2021). In polarized epithelial MDCK monolayers, Vps41 KO blocks a key early step of ciliogenesis in which cortical actin is remodeled and cleared around the apically docked basal body (Hoffman and Prekeris, 2022; Jewett et al., 2021). The canonical role of the HOPS complex is as a membrane tether in lysosomal trafficking (Balderhaar and Ungermann, 2013; van der Beek et al., 2019). It was unclear whether the requirement for HOPS in ciliogenesis reflected a requirement for lysosomal trafficking in ciliogenesis, or whether it represented a separate function of the HOPS complex. It was also unknown whether HOPS participates directly in actin clearing and ciliogenesis at the basal body, or whether its role is indirect. Addressing these outstanding questions is the main focus of this study.

### Requirement for lysosomal trafficking in ciliogenesis

We reasoned that if the actin-clearing and ciliogenesis role of HOPS were a consequence of its role in lysosomal fusion, then treatment with CQ, an inhibitor of lysosomal fusion (Mauthe et al., 2018; Mullock et al., 1998), should recapitulate the actin-clearing and ciliogenesis defects of Vps41 KO. The effects of CQ on LE/Ls in MDCK cells were similar to the effects of Vps41 KO, producing enlarged LEs swollen with undegraded cargo (Fig. 1A,B). Actin clearing and ciliogenesis were blocked in CQ-treated MDCK as in Vps41 KO MDCK cells (Fig. 1C–G; Fig. S1), suggesting that those phenotypes were indeed due to disruption of lysosomal fusion, rather than a separate non-lysosomal function of Vps41. In both Vps41 KO and CQ-treated cells, actin-clearing defects were accompanied by defects in exclusion of non-ciliary membrane proteins (Fig. S2A) and appeared to be upstream of ciliation defects (Fig. S2B–D), emphasizing the importance of the actin-clearing step for ciliogenesis in polarized epithelia.

These results align with some previous literature linking lysosomal fusion to ciliogenesis. For example, two lipid phosphatases – INPP5E, which is among the genes mutated in the ciliopathy Joubert syndrome, and OCRL1, mutated in the ciliopathy-like Lowe syndrome – have been reported to be important both for primary ciliogenesis (Hardee et al., 2017; Luo et al., 2012a,b; Xu et al., 2017) and for autophagosome–lysosome fusion (De Leo et al., 2016; Hasegawa et al., 2016). In a zebrafish model of INPP5E-related Joubert syndrome, renal ciliogenesis defects were accompanied by defects in apical cortical actin organization (Xu et al., 2017), potentially reflecting a commonality with the actin cortical-clearing defects described in this paper. Depletion of VAMP7, a SNARE protein involved in lysosomal fusion, was also shown to disrupt ciliation in MDCK cells, whereas depletion of the lysosomal enzyme α-gal A did not affect ciliation (Szalinski et al., 2014), which might suggest that it is specifically the fusion step of lysosomal trafficking, rather than lysosomal function, that is important to ciliogenesis.

Although these results demonstrated a requirement for lysosomal fusion during ciliogenesis, what remained unclear was the mechanism by which lysosomal fusion affects cilia formation. Several studies have shown that certain proteins that inhibit ciliogenesis, including OFD1 (Tang et al., 2013), MYH9 (Yamamoto et al., 2021) and CP110 (Liu et al., 2021), are degraded by selective autophagy to promote ciliogenesis. Given that autophagy involves HOPS-dependent autophagosome–lysosome fusion (Jiang et al., 2014), we hypothesized that the ciliogenesis defects of Vps41 KO MDCK cells might be due to failure to degrade these ciliogenesis inhibitors. However, although autophagy was impaired in Vps41 KO cells (Fig. 2A,B), and although treatment with the early-stage autophagy inhibitor MRT68921 also mimicked the actin-clearing and ciliogenesis defects of Vps41 KO (Fig. 2C), we found that Vps41 KO cells did not show excess accumulation of MYH9 (Fig. 2D–F), OFD1 or CP110 (Fig. 2D,E). We also did not observe MYH9 accumulation upon treatment with autophagy inhibitor BafA (Fig. 2F), suggesting that MYH9 was not an autophagy cargo in polarized MDCK cells. The difference between these results and those of previous studies (Liu et al., 2021; Tang et al., 2013; Yamamoto et al., 2021) might reflect differences between starvation-induced and starvation-independent ciliogenesis. Serum starvation is commonly used to induce ciliogenesis in cultured cells, especially in non-polarized cells. However, serum starvation also induces autophagy, and it was under starvation conditions that autophagy of MYH9, OFD1 and CP110 was shown to be important for ciliogenesis (Liu et al., 2021; Pierce and Nachury, 2013; Tang et al., 2013; Yamamoto et al., 2021). In contrast, our study examined ciliogenesis in polarized MDCK monolayers in complete medium. Thus, selective autophagy of ciliogenesis inhibitors might be important for starvation-induced ciliogenesis in some cell types, but not for starvation-independent ciliogenesis in polarized epithelial cells.

### Disruption of lysosomal trafficking disrupts recruitment of Rab19 to the site of ciliogenesis

In searching for alternative explanations for the actin-clearing and ciliogenesis defects of Vps41 KO MDCK, we investigated Rab19, as previous work had shown that Rab19 drives actin clearing during ciliogenesis and that the HOPS complex is a Rab19 effector (Jewett et al., 2021). As previously reported (Jewett et al., 2021), in WT MDCK monolayers, Rab19 localized to the actin cortical clearing around the basal body and only occasionally localized to LE/Ls (Fig. 3A–D; Fig. S3A). Rab19 was also observed in the Golgi (Fig. S4A,B), suggesting that the role of Rab19 in ciliogenesis might involve transporting some ciliogenesis factors from the Golgi to the basal body. In Vps41 KO or CQ-treated cells, however, Rab19 accumulated on enlarged LEs, and its recruitment to the basal body and Golgi was reduced (Fig. 3A–D; Figs S3A, and S4A,B).

In support of a hypothesis that depletion of Rab19 from the basal body contributes to the actin-clearing and ciliogenesis defects of Vps41 KO and CQ-treated cells, we found that GFP–Rab19 overexpression rescued those defects (Fig. 3E,F; Fig. S3B–D). Thus, retention of Rab19 on LEs in Vps41 KO and CQ-treated cells reduces the fraction of cellular Rab19 that can be recruited to the basal body, thereby disrupting actin clearing and ciliogenesis (Fig. 7). GFP–Rab19 overexpression only partially rescues ciliation in Vps41 KO cells, whereas it fully rescues the phenotype in CQ-treated cells (Fig. 3E,F; Fig. S3D), suggesting that in Vps41 KO cells a reduction of Rab19 at the basal body contributes to but is not the sole reason for ciliation defects. It might be that HOPS complex depletion also affects the localization of other, as yet unidentified, ciliogenesis regulators. Rab19 targeting to the apical cortex was also lost in cells treated with autophagy inhibitor MRT68921 (Fig. S3E). Thus, the ciliogenesis defect in MRT68921-treated cells (Fig. 2C) – and potentially some of the ciliogenesis defects associated with other conditions that disrupt autophagy (Morleo and Franco, 2019; Yamamoto and Mizushima, 2021) – might also be due to depletion of Rab19 from the site of ciliogenesis.

### Cell type dependence of relationship between lysosomal fusion and ciliogenesis

Because some aspects of ciliogenesis differ between the extracellular pathway described in polarized epithelial cells and the intracellular pathway described in other cell types (Hoffman and Prekeris, 2022; Molla-Herman et al., 2010; Sorokin, 1968), we tested the cell type dependence of the interrelationship between lysosomal fusion, Rab19 localization and ciliogenesis. The effects of CQ that we had observed in MDCK cells were largely conserved in another renal polarized epithelial cell line, mIMCD3 (Fig. 4). Rab19, which was enriched at the base of cilia in untreated mIMCD3 cells (Fig. 4A), became aberrantly accumulated on LE/Ls in CQ-treated mIMCD3 (Fig. 4A,B), and ciliogenesis was blocked (Fig. 4A,C).

In non-polarized RPE1 cells, however, the effect was not the same (Fig. 5). Although Rab19 localization to LE/Ls was increased in CQ-treated RPE1 cells (Fig. 5A), ciliogenesis appeared unaffected (Fig. 5B–E). The mechanism by which impaired lysosomal fusion disrupts ciliogenesis by disrupting Rab19 recruitment to the site of ciliogenesis thus might be specific to polarized epithelial cells and to the extracellular ciliogenesis pathway. This is surprising because Rab19 KO reduces ciliogenesis in RPE1 as well as MDCK cells (Jewett et al., 2021). One possibility is that, although Rab19 is important for ciliogenesis in both RPE1 and MDCK cells, RPE1 cells (unlike MDCK) might have sufficient excess of Rab19 so that its accumulation on LE/Ls does not deplete the pool of Rab19 at the basal body below the threshold needed for ciliogenesis. Cortical actin is more prominent in MDCK than in RPE1 cells (compare Figs 1C and 5C), so the amount of cortical actin that has to be cleared during ciliogenesis might be greater in polarized epithelial cells, perhaps requiring a higher threshold of Rab19 at the basal body to mediate this function. Further studies will be needed to elucidate determinants of this cell type dependence, which could be relevant to understanding the tissue-specific manifestations of ciliopathies.

### Rab19 function at LEs

The observation that Rab19 accumulates on LEs when lysosomal fusion is inhibited (Figs 3C,D, 4A,B and 5A; Fig. S3A,) suggested that Rab19 might have a previously uncharacterized function at the LE. Rab19 KO did not affect LE/L size, but expression of the Rab19-CA mutant exacerbated the enlargement of LEs upon CQ treatment (Fig. S4C,D). A potential explanation is that Rab19 mediates the transport of certain cargoes to LEs, which under normal conditions would only constitute a minor fraction of total LE cargo volume, but when downstream lysosomal degradation is blocked then an increase in delivery of these Rab19 cargoes results in increased swelling of the LEs. Further studies will be needed to identify these Rab19-dependent LE cargoes.

### Differential roles of the LE/L-associated HOPS complex and EE-associated CORVET complex in ciliogenesis

We investigated the interaction between Rab19 and HOPS, aiming to determine which subunit of the HOPS complex was responsible for binding Rab19. Surprisingly, we found that the HOPS–Rab19 interaction – unlike the previously characterized interaction of HOPS with Rab7 via the HOPS-specific subunits Vps41 and Vps39 (Bröcker et al., 2012) – appears to be mediated by the core complex (Vps11, Vps16, Vps18 and Vps33A) that is shared between the LE/L-associated HOPS and EE-associated CORVET complexes (Fig. S5). This revealed that Rab19 might interact with CORVET on EEs as well as with HOPS on LE/Ls, leading us to probe the respective roles of EEs and LE/Ls in ciliogenesis.

The original work that identified HOPS as being required for ciliogenesis (Jewett et al., 2021) left open the question of whether HOPS is actually localized to the basal body to participate directly in ciliogenesis. Immunostaining for the HOPS/CORVET shared core subunit Vps11 showed that Vps11-positive vesicles were frequently present at the basal body (Fig. 6A,B,D). Using LysoTracker as a marker for LE/Ls and EEA1 immunostaining as a marker for EEs, we found that EEs were often observed at the basal body and locations of actin clearing, whereas LE/Ls were not (Fig. 6B,D,F,G). Thus, it appears that EE-associated CORVET rather than LE/L-associated HOPS is present at the site of ciliogenesis.

### Conclusions and biomedical implications

Collectively, our observations favor a model in which the HOPS complex is not directly involved in ciliogenesis at the basal body (Fig. 7). Instead, HOPS-mediated lysosomal fusion occurring elsewhere in the cell maintains the flow of endolysosomal trafficking, which is needed to balance Rab19 activity between the separate functions of Rab19 in ciliogenesis and in LE cargo transport. Under normal conditions in polarized epithelial cells (Fig. 7A), a fraction of Rab19 is transiently recruited to LEs and cycles off of these membranes after lysosomal fusion, keeping a large pool of Rab19 available to drive actin clearing and ciliogenesis at the basal body (where it likely recruits a negative regulator of Rho–ROCK–actomyosin contractility to facilitate actin clearing, and also regulates vesicular delivery of ciliary membrane, as suggested by Jewett et al. (2021). When lysosomal fusion is impaired, as in Vps41 KO or CQ-treated cells, then Rab19 is abnormally retained on LEs, which fail to fuse to lysosomes; this depletes the pool of Rab19 available to be recruited to the basal body, and thereby disrupts Rab19-mediated actin clearing and ciliogenesis (Fig. 7B).

These results highlight a relatively unexplored way that defects in lysosomal trafficking can disrupt other cellular processes such as ciliogenesis – besides blocking the degradation of lysosomal cargoes, such defects also dysregulate the activity of cellular machinery that is normally shared between endolysosomal functions and other functions. Although we focused here on the mislocalization of Rab19 and its effect on ciliogenesis, it would be interesting for future studies to investigate what other factors are abnormally retained on LE/Ls when their fusion is impaired and what other cellular functions might be dysregulated as a result.

This mechanism (i.e. mislocalization of Rab19 leading to defects in ciliogenesis) might be relevant to certain ciliopathies – e.g. INPP5E-associated Joubert syndrome, as discussed above – and other conditions that perturb lysosomal trafficking. For mutations in Vps41 and other HOPS subunits, the main disease manifestations are neurological disorders often involving dystonia (Monfrini et al., 2021a,b; Sanderson et al., 2021; Steel et al., 2020; van der Welle et al., 2021). These HOPS mutations cause lysosomal abnormalities in fibroblasts (Monfrini et al., 2021a; Steel et al., 2020; van der Welle et al., 2021), but the mechanism linking those lysosomal defects to the dystonia phenotype is unclear. Investigating whether Rab19 is mislocalized and whether ciliogenesis is impaired by these HOPS mutations could inform potential treatments for HOPS-associated neurological disorders. Moreover, given that Rab19 targeting to the site of ciliogenesis was also disrupted when autophagy initiation was blocked (Fig. S3E), it would be worth investigating the role of Rab19 in disorders where ciliary defects are associated with abnormal autophagy, including polycystic kidney disease (Morleo and Franco, 2019; Wang and Dong, 2020; Zhu et al., 2016) and focal cortical dyslamination (Morleo and Franco, 2019; Park et al., 2018). Besides genetic diseases, other conditions can also impair lysosomal fusion and thus might impact cilia by the mechanism outlined here. For example, CQ and the related lysosomal-fusion-inhibiting drug hydroxychloroquine (HCQ) (Mondal et al., 2022) are used to treat malaria and certain autoimmune diseases; these drugs can cause adverse effects including neuropsychiatric events, cardiotoxicity and retinopathy (Doyno et al., 2021; Schrezenmeier and Dörner, 2020). In light of our results showing that CQ blocks ciliogenesis, it will be important to assess whether cilia are altered in patients treated with CQ or HCQ, as cilia dysfunction could contribute to adverse effects of these drugs. Thus, the indirect role of lysosomal trafficking in regulating Rab19 activity for ciliogenesis is potentially relevant to a variety of diseases ranging from ciliopathies to malaria.

## MATERIALS AND METHODS

### Cell lines and cell culture

The MDCK, mIMCD3, RPE1 and HEK293T cell lines were obtained from ATCC (Manassas, VA, USA); details of these cell lines are listed in Table S1. Cells were maintained at 37°C with 5% CO_2_ in complete medium. For MDCK, RPE1 and HEK293T cells, the complete medium consisted of DMEM (#10-017-CV, Corning, Corning, NY, USA) with 10% fetal bovine serum (FBS) (#PS-300, Phoenix Scientific, San Marcos, CA, USA) and 1× penicillin-streptomycin (#30-002-CL, Corning). For mIMCD3 cells, the complete medium consisted of DMEM/F12 (#SH30023.01, Cytiva, Marlborough, MA, USA) with 10% FBS and 1× penicillin-streptomycin.

### CRISPR knockout lines and overexpression stable cell lines

The generation of a parental MDCK line stably expressing Tet-inducible Cas9, and the generation of the Vps41 and Rab19 CRISPR KO lines on that background, was previously described (Jewett et al., 2021). The control cell line referred to as ‘WT MDCK’ throughout the manuscript is the Tet-inducible Cas9 parental line, to provide an appropriately matched control for the CRISPR knockout lines. The cell lines labeled as Vps41 KO#1 and Vps41 KO#2 are two clonal lines of Vps41 KO MDCK cells.

The MDCK lines stably overexpressing GFP–Rab19 on the WT or Rab19 KO background, and the RPE1 lines stably overexpressing GFP-Rab19 on the Rab19 KO background or pH-Smo on the WT background, were previously described (Jewett et al., 2021). The MDCK lines stably overexpressing GFP–Rab19 on the Vps41 KO background and GFP–MYH9 on the WT or Vps41 KO background, and the mIMCD3 line stably overexpressing GFP–Rab19 on a WT background, were generated by the same approach, using lentiviral transduction and puromycin selection.

### Drug treatments

For CQ treatment, chloroquine diphosphate salt (#200-055-2, Sigma, St. Louis, MO, USA) was added to the medium at a concentration of 10 μM for MDCK or RPE1 cells or 100 μM for mIMCD3 cells, beginning ∼0.5–2 h after seeding the cells for the experiment, and refreshed daily until fixation (2–3 days for MDCK experiments, as specified in the figure legends; 3 days for RPE1 experiments; 4–5 days for mIMCD3 experiments, as specified in the figure legends). For MRT68921 treatment, MRT68921 dihydrochloride (#HY-100006A, MedChemExpress, Monmouth Junction, NJ, USA) was added to the medium at 600 nM concentration, beginning 0.5–1 h after seeding the cells for the experiment, and refreshed daily until fixation (3 days). For BafA treatment, bafilomycin A1 (#S1413, Selleck Chemicals, Houston, TX, USA) was added to the medium at 100 nM concentration for the final 16 h prior to fixation.

### Plasmids

The pLVX-GFP-Rab19 lentiviral transfer plasmid used to establish the GFP–Rab19 stable cell lines was previously described (Jewett et al., 2021). A pLVX-GFP-MYH9 transfer plasmid was cloned by ligating a NdeI/SalI-digested GFP-MYH9 cassette from CMV-GFP-NMHC II-A (Addgene plasmid #11347, deposited by Robert Adelstein) into NdeI/XhoI-digested pLVX-Puro vector. For lentiviral vector production, the transfer plasmid was co-transfected with the packaging plasmid pΔ8.9 and pseudotyping plasmid pVSV-G into HEK293T cells.

The pGEX-KG-Rab19 plasmid, encoding GST-tagged Rab19 for expression in *E. coli* for recombinant protein production, was previously described (Jewett et al., 2021). The HA-Vps3 expression plasmid was a gift from Dr Jacques Neefjes (Cell & Chemical Biology, LUMC, The Netherlands), and the Vps8–GFP expression plasmid was a gift from Dr Judith Klumperman (Cell Biology, UMC Utrecht, The Netherlands).

### Antibodies and dyes

Antibodies and dyes used for immunofluorescence and western blotting experiments are listed in Table S2.

### Western blotting

For western blotting of cell lysates, MDCK samples cultured at high confluency were washed with phosphate-buffered saline (PBS) and lysed in a buffer of Tris-buffered saline (TBS) with 1% Triton X-100 and 1 mM EDTA for 30 min on ice, then centrifuged for 20 min at 15,000 ***g*** at 4°C. The supernatants of the cell lysates were normalized for total protein concentration according to the Bradford assay (#5000006, Bio-Rad, Hercules, CA, USA), mixed with SDS loading dye, and boiled for 5–10 min at 95°C. The samples were then run on SDS-PAGE gels, transferred to PVDF membranes, blocked for at least 20 min in a buffer consisting of Intercept (TBS) Blocking Buffer (#927-60001, LI-COR) diluted 1:3 in TBS with 0.05% Tween-20 (TBST), probed with the primary antibodies diluted in blocking buffer for at least 2 h at room temperature or overnight at 4°C, washed four times for ≥5 min with TBST, probed with the secondary antibodies diluted in blocking buffer for 1-2 h at room temp, washed four times for ≥5 min with TBST and once with TBS, and then imaged on an Odyssey DLx imaging system (LI-COR).

Densitometry of the blot images was performed in Image Studio Lite Version 5.2.5 (LI-COR) using median background subtraction. Detected levels of the target protein were normalized according to a tubulin loading control, then normalized to the level in detected in the WT MDCK control in the same experiment. Full images of western blots shown in this paper are presented in Fig. S7.

### GST–Rab19 pulldown assays

GST-tagged Rab19 protein was recombinantly produced in BL21 Codon Plus *E. coli*, purified using glutathione agarose beads and eluted with free glutathione as previously described (Jewett et al., 2021).

The GST–Rab19 protein was used for glutathione bead pulldown assays with cell lysates as previously described (Jewett et al., 2021; Willenborg et al., 2011). Cells were lysed in a buffer of 20 mM HEPES, pH 7.4, 150 mM NaCl, 1% Triton-X, 1 mM PMSF and EDTA-free protease inhibitor cocktail (Roche). Purified GST–Rab19 protein, or free GST protein as a negative control, was added to the cell lysates. For GTP/GDP loading, the GST–Rab19 and lysate mixtures were incubated with 5 mM EDTA for 10 min, then 5 mM GMP-PCP (non-hydrolyzable GTP analog; #AB146660-1001, Abcam) or 5 mM GDP (#AB146529-1001, Abcam) for 10 min, and then 15 mM MgCl_2_ for 10 min. The mixtures were incubated with glutathione beads for at least 1 h while rotating, followed by washes with a buffer of 20 mM HEPES, pH 7.4, 300 mM NaCl, 0.1% Triton-X to remove unbound proteins, and then bound proteins were eluted from the beads by boiling for 10 min at 95°C in 1× SDS loading dye. The bead eluates and cell lysates were then analyzed by SDS-PAGE and western blotting as described above.

### siRNA knockdowns of HOPS subunits

The siRNAs (listed in Table S3) were purchased from Qiagen (Germantown, MD, USA) and were transfected into HEK293T cells using Lipofectamine RNAiMAX transfection reagent (#13778075, Thermo Fisher Scientific), and samples were harvested 2 days post transfection. For RT-qPCR validation of the siRNA knockdowns, RNA was isolated using TRIzol reagent (#15596026, Thermo Fisher Scientific), cDNA was synthesized using the SuperScript IV First-Strand Synthesis System (#18091050, Thermo Fisher Scientific), and qPCR was performed using iTaq Universal SYBR Green Supermix (#1725121, Bio-Rad), with primers (Table S3; purchased from Integrated DNA Technologies, Coralville, IA, USA) designed to span an exon–exon junction to prevent amplification from genomic DNA, and analyzed using a StepOnePlus Real-Time PCR machine (#4376600, Thermo Fisher Scientific). GAPDH was analyzed as a housekeeping gene control.

### Immunofluorescence experiments

#### Culturing of MDCK and mIMCD3 cells as polarized monolayers on Transwell filters

For MDCK and mIMCD3 immunofluorescence experiments, 0.4 µm pore PET membrane Transwell filter inserts (12-well filters, #665640, Greiner Bio-One, Monroe, NC, USA, or #38023, STEMCELL Technologies, Vancouver, BC, Canada; or 24-well filters, #662640, Greiner Bio-One) in wells of tissue-culture well plates were pre-coated with rat-tail collagen and cured under UV light for ≥30 min prior to use. MDCK or mIMCD3 cells were plated on the filters at a highly confluent density of 2.2×10^5^ cells/cm^2^ (2.5×10^5^ cells per 12-well filter, or 7.4×10^4^ cells per 24-well filter). The cells were cultured on the filters, in complete medium, which was changed daily, for 3 days prior to fixation for MDCK experiments assessing ciliation, actin cortical clearing and basal body localization phenotypes, 2 days for some of the other MDCK experiments, such as those assessing LE/L phenotypes, or 4–5 days (as specified in the figure legend) for the mIMCD3 ciliation experiments.

#### Culturing of RPE1 cells on coverslips

For RPE1 immunofluorescence experiments, 25×25 mm #1 glass coverslips 25×25 mm (#48366-067, VWR, Radnor, PA) were placed in wells of six-well tissue-culture well plates, pre-coated with rat-tail collagen for ≥20 min, and cured under UV light for ≥1 h prior to use. 2.5×10^5^ RPE1 cells were plated per coverslip, in complete medium. The following day, the complete medium was replaced with serum-starvation medium consisting of DMEM with only 0.5% FBS and 1× penicillin-streptomycin, and serum-starvation was continued for 48 h prior to fixation.

#### Immunostaining and confocal fluorescence microscopy

For experiments with LysoTracker, the live cells were stained with LysoTracker Red DND-99 (#L7528, Thermo Fisher Scientific) at 500 nM (1:2000 dilution) in the medium for the final 30 min prior to fixation. Any experimental treatments (e.g. CQ or MRT68921 and serum-starvation on RPE1 samples) were continued during LysoTracker staining.

The following immunostaining protocol was performed at room temperature. Cells were fixed with 4% paraformaldehyde (#15710, Electron Microscopy Sciences, Hatfield, PA, USA) in PBS for 20 min, quenched twice for 5 min with 0.1 M glycine in PBS and rinsed with PBS. For MDCK or mIMCD3 samples on Transwell filters, the filters were then cut out of the Transwell inserts and placed on parafilm in a humidified dish for the following steps: for RPE1 samples on coverslips, the entire staining protocol was performed on the coverslips in six-well-plate wells. Samples were rinsed with PBS with 0.1% Triton X-100 (PBST), blocked with 10% normal donkey serum (#017-000-121, Jackson ImmunoResearch) in PBST for ≥1 h, and stained overnight with primary antibodies (Table S3) diluted in blocking buffer. The next day, the samples were washed 5×≥5 min with PBST, stained with Hoechst 33342 and dye-labeled secondary antibodies (Table S3) and/or phalloidin for 2 h, and washed 5×≥5 min with PBST and once with PBS, then mounted on slides (#TNR WHT90, Tanner Scientific, Sarasota, FL, USA) with Vectashield (#H-1000, Vector Laboratories, Newark, CA), filters were covered with coverslips (#48366-067, VWR), and the coverslips were sealed to the slides with clear nail polish. Confocal fluorescence microscopy images were acquired either on a Nikon A1R confocal microscope (Nikon, Tokyo, Japan) using a 60× oil objective and NIS-Elements AR software (Nikon), or on a Leica SP8 confocal microscope (Leica Microsystems, Wetzlar, Germany) using a 63× oil objective and LAS X software (Leica).

### Quantitative image analysis

#### LysoTracker compartment size analysis

*Z*-stack images of MDCK monolayers (grown for 2 days on filters, and stained with LysoTracker) were acquired on the Nikon A1R confocal microscope, with 0.21 µm pixel size and z-step. Analysis of LE/L size was performed in FIJI (Schindelin et al., 2012) as follows: 50 µm×50 µm ROIs were cropped from the LysoTracker Red channel image stack. The image stack was smoothed, a maximum intensity projection (MIP) was taken, and rolling-ball background subtraction was applied with a radius of 20 pixels. The image was then binarized using the Yen thresholding algorithm. Particle analysis was applied to quantify the size of LysoTracker compartments, analyzing particles with size ≥3 pixels and circularity 0.50–1 and excluding those on edges. Between six and eight technical replicates (ROIs) were averaged from each biological replicate for each condition. Outlier biological replicates were excluded (ROUT, Q=2%).

#### Measuring apical cortical actin clearing

*Z*-stack images of MDCK monolayers (grown for 3 days on filters, and stained with anti-γ-tubulin antibody and phalloidin) were acquired on the Nikon A1R confocal microscope, with 0.28 µm pixel size and *z*-step. Analysis of apical actin cortical clearing was performed in FIJI, as follows. The z-stack was resliced to obtain the side view. Individual basal bodies (marked by γ-tubulin) docked at the apical actin cortex (marked by phalloidin) were selected for analysis. An 8 µm wide ×5 µm high ROI was drawn centered around the selected basal body. The Plot Profile function was used to measure the vertically averaged pixel intensities in the γ-tubulin and phalloidin channels along the horizontal distance through the ROI. Each intensity profile was normalized to have mean=1, by dividing each intensity value by the mean of that entire profile. To determine the fraction of cortical actin remaining over the basal body, the mean of the values in the actin intensity profile within 0.3 µm horizontal distance from the center of the ROI was divided by the mean of the values in the actin intensity profile between 2 µm and 4 µm from the center of the ROI. Between ten and 15 technical replicates (individual basal bodies) were averaged from each biological replicate for each condition.

#### Measuring ciliation

For quantification of MDCK ciliation, *Z*-stack images of MDCK monolayers (grown for 3 days on filters prior to fixation, and stained with Arl13b antibody and Hoechst 33342) were acquired on the Nikon A1R confocal microscope, with a 0.28 µm pixel size and z-step. Analysis of the percentage of cells exhibiting an Arl13b-positive primary cilium was performed in FIJI, as follows: To count cilia, a MIP of the Arl13b channel stack was taken, and rolling-ball background subtraction was applied with a radius of 100 pixels. The image was binarized using the Shanbhag thresholding algorithm, and particle analysis was applied to quantify the number of Arl13b puncta, analyzing particles with size ≥2 pixels. To count cells, a MIP of the Hoechst 33342 channel stack was taken, the image was smoothed, and rolling-ball background subtraction was applied with a radius of 100 pixels. The image was binarized using the MinError(I) thresholding algorithm in the Auto Threshold plugin, and watershed separation was used to distinguish overlapping nuclei. Particle analysis was applied to quantify the number of nuclei, analyzing particles with size ≥150 pixels. The number of cilia was then divided by the number of nuclei to calculate the percent ciliation. A total of three or four technical replicates (142 µm×142 µm fields of view) were averaged from each biological replicate for each condition.

For quantification of RPE1 ciliation, RPE1 samples (grown for 3 days on coverslips with serum starvation for the final 2 days prior to fixation) were stained and imaged in the same manner as for MDCK cells, and the percentage of cells exhibiting a primary cilium was analyzed by the same method as above, except with the following changes: the MIP of the Arl13b channel was binarized using the Yen thresholding algorithm in the Auto Threshold plugin, the minimum particle size for cilia was set at 5 pixels, the count of cell nuclei was corrected by manual counting in cases where the automated segmentation of nuclei did not perform well, and eight technical replicates were averaged from each biological replicate for each condition.

#### Quantifying Rab19 enrichment at the basal body

*Z*-stack images of GFP–Rab19-expressing MDCK monolayers (grown for 3 days on filters, and stained with anti-γ-tubulin antibody and phalloidin) were acquired on the Leica SP8 confocal microscope, with 0.12 µm pixel size and *z*-step, spanning from above the apical cortex to a mid-basolateral plane of the monolayer. Analysis of the enrichment of Rab19 vesicles at the basal body was performed in FIJI, as follows: The z-stack was resliced to obtain the side view. Rolling ball background subtraction was applied with a radius of 75 pixels. Side view slices showing apically docked basal bodies were selected for analysis, and GFP-Rab19 signal in the image slice was binarized using the Yen thresholding algorithm. The binarized result was visually inspected to check that it was a reasonable representation of the Rab19 compartments in the original image. Any cells for which almost all pixels were thresholded as either uniformly positive or uniformly negative (which could occur when GFP–Rab19 expression in the cell of interest was far brighter or far dimmer than in the other cells in the slice) were excluded from analysis. A ‘basal body’ ROI of 3 µm×2 µm was then drawn with its upper edge centered at the apical end of the basal body, and a ‘cytosol’ ROI was drawn freehand to enclose all of the cytosol of the selected cell that was visible in the image slice, bounded by the phalloidin-stained actin cortex and excluding the nucleus. The density of Rab19-positive pixels (number of positive pixels divided by total pixel area of the ROI) in the basal body ROI was divided by the density of Rab19-positive pixels in the cytosol ROI to obtain a measure of Rab19 enrichment at the basal body. Ten technical replicates (individual cells) were averaged from each biological replicate for each condition.

#### Analyzing Rab19 colocalization with LysoTracker

*Z*-stack images of GFP–Rab19-expressing MDCK monolayers (grown for 2 days on filters and stained with LysoTracker) were acquired on the Leica SP8 confocal microscope, with 0.12 µm pixel size. Background subtraction and binarization of the GFP–Rab19 and LysoTracker image channels was performed in FIJI, as follows: rolling ball background subtraction was applied with a radius of 100 pixels. 30 µm×30 µm ROIs were cropped from the image stack, and a single slice at a mid-apical level of the monolayer was selected for analysis. The image slice was binarized using the Yen thresholding algorithm. The fractional overlap (Manders' colocalization coefficients; Manders et al., 1993) between GFP–Rab19 and LysoTracker was then computed using a custom script written in MATLAB (MathWorks, Natick, MA, USA; available upon request). Four technical replicates (ROIs) were averaged from each biological replicate for each condition.

#### Quantifying EEA1 and LysoTracker enrichment at the basal body

*Z*-stack images of MDCK monolayers (grown for 3 days on filters and stained with anti-γ-tubulin and anti-EEA1 antibody and/or LysoTracker) were acquired on the Leica SP8 confocal microscope, with a 0.12 µm pixel size and *z*-step, spanning from above the apical cortex to a mid-basolateral plane of the monolayer. Analysis of the enrichment of EEA1 and/or LysoTracker vesicles at the basal body was performed by the same method as described above for analysis of Rab19 enrichment at the basal body. A total of 15 technical replicates (individual cells) were averaged from each biological replicate for each condition.

#### Analyzing Vps11 colocalization with EEA1 and with LysoTracker

*Z*-stack images of MDCK monolayers (grown for 3 days on filters and stained with anti-Vps11 antibody and either anti-EEA1 antibody or LysoTracker) were acquired on the Leica SP8 confocal microscope, with a 0.12 µm pixel size. Background subtraction and binarization, followed by computation of the fractional overlap in a selected z-slice at a mid-apical level of the monolayer, was performed by the same methods as described for Rab19 and LysoTracker colocalization above.

### Statistical analysis

GraphPad Prism 9.5.0 software (GraphPad Software, Boston, MA, USA) was used to generate graphs and to calculate *P*-values. For all of the quantitative image analysis experiments, each data point on the graphs in the figures represents the mean of the technical replicates (ROIs or individual cells) for one biological replicate (separate experiment), except as noted for the graph in Fig. S2B where the data points represent individual technical replicates (individual cells). *P*-values were calculated from the means for the biological replicates. For the analysis of MDCK percentage ciliation, statistical significance was assessed by mixed-effects analysis with Geisser–Greenhouse correction and Šidák's multiple comparisons test. For the analysis of RPE1 percentage ciliation, statistical significance was assessed by Wilcoxon test. For the analysis of basal-body enrichment of EEA1 and LysoTracker, statistical significance was assessed by Kolmogorov–Smirnov test. For the other quantitative image analysis experiments, statistical significance was assessed by Brown–Forsythe and Welch ANOVA test and Dunnett's T3 multiple comparisons test. For the western blot densitometry results, each data point on the graphs represents the result from one biological replicate (separate set of cell lysates), normalized to the loading control and then to the WT level of the target protein from the same biological replicate, and statistical significance was assessed by one-sample *t*-test.

## Supplementary Material

10.1242/joces.261047_sup1 Supplementary information
