# Supplementary material for: HOPS-dependent lysosomal fusion controls Rab19 availability for ciliogenesis in polarized epithelial cells
Source: J Cell Sci. 2023 Sep 4;137(5):jcs261047. doi: 10.1242/jcs.261047 (PMC10499034; doi:10.1242/jcs.261047)
Supplement: Supplementary information [file joces-137-261047-s1.pdf]

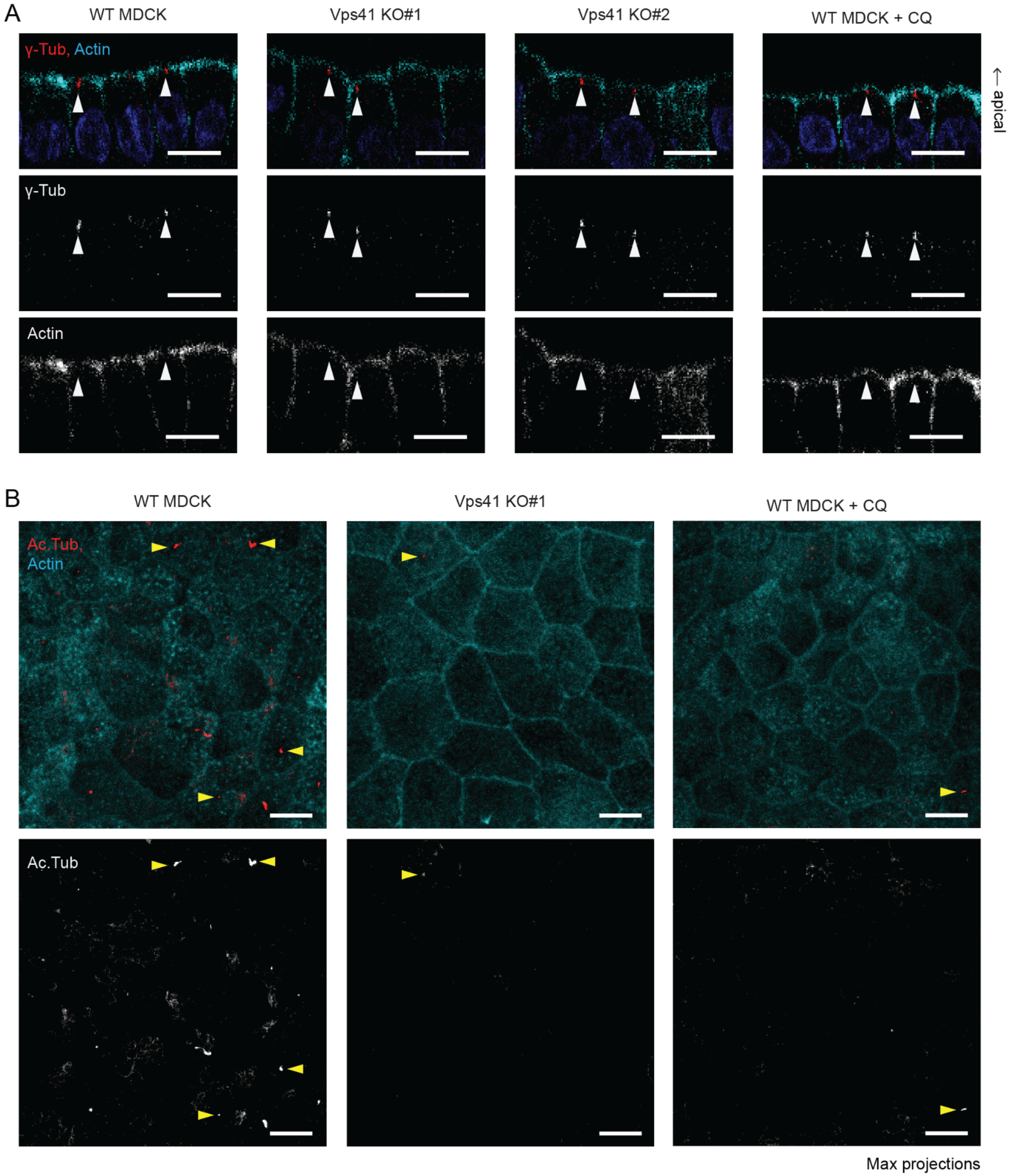

Journal of Cell Science • Supplementary information

Fig. S1. Additional data on actin-clearing and ciliation defects of Vps41 KO and CQ-treated MDCK.

A. WT, Vps41 KO, or CQ-treated (10 μM for 3 days) MDCK cells, stained with γ-tubulin antibody andPhalloidin. Side views, scale bars 10 μm. Arrows: basal bodies. WT MDCK cells exhibit a clearing in the apical actin cortex proximal to the basal body, and this actin cortical clearing is impaired in both Vps41 KO and CQ-treated cells.

B. WT, Vps41 KO, or CQ-treated (10 μM for 3 days) MDCK cells, stained with acetylated α-tubulin antibody and Phalloidin. MIPs, scale bars 10 μm. Arrows: examples of cilia. Ciliation is impaired in both Vps41 KO and CQ-treated cells.

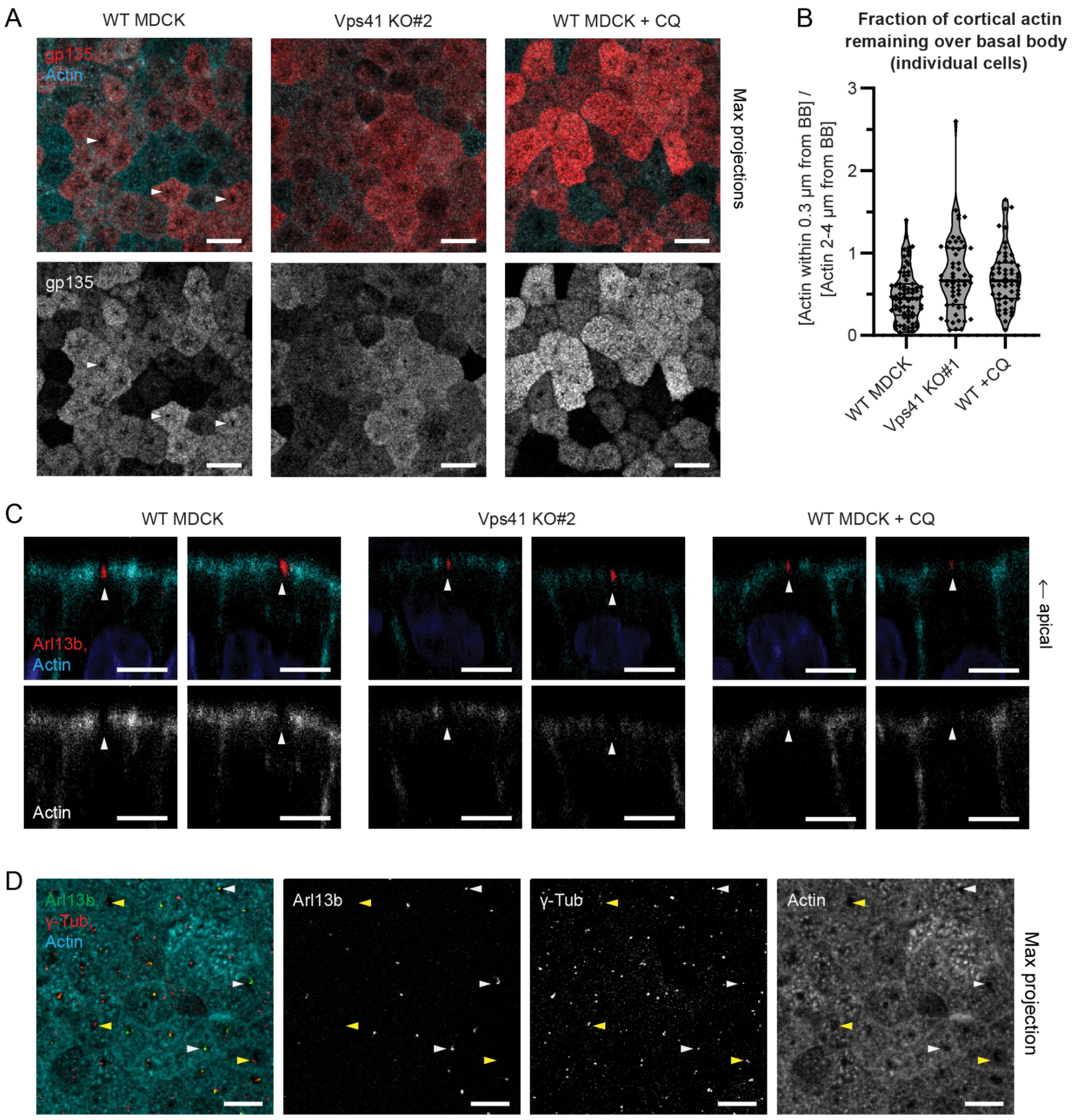

Fig. S2. Gp135 cortical clearing and primary ciliogenesis are dependent on actin cortical clearing.

A. WT, Vps41 KO, or CQ-treated (10 μM for 3 days) MDCK cells, stained with gp135 antibody and Phalloidin; MIPs, scale bars 10 μm. In WT MDCK, the non-ciliary apical membrane protein gp135 is excluded from the site where apical cortical actin is cleared (examples indicated with arrows). In both Vps41 KO and CQ-treated cells, the lack of a clearing in the actin cortex is accompanied by the lack of an exclusion zone in gp135.

Journal of Cell Science • Supplementary information

B. Fraction of cortical actin remaining above the basal body for all individual cells analyzed, of which the means of each biological replicate are shown in Fig. 1E. Points represent individual cells; bars represent median and quartiles.

C. WT, Vps41 KO, or CQ-treated (10 μM for 3 days) MDCK cells, stained with Arl13b antibody and Phalloidin.

Side views, examples selected to show cilia (arrows) although cilia are infrequent overall in Vps41 KO and CQ-treated cells. Scale bars 5 μm. In all three conditions, when a cilium is present, it is associated with a clearing in the apical actin cortex.

D. WT MDCK cells stained with Arl13b antibody, γ-tubulin antibody, and Phalloidin; MIPs, scale bars 10 μm. White arrows indicate examples of cilia in actin clearings, and yellow arrows indicate examples of actin clearings without cilia.

Journal of Cell Science • Supplementary information

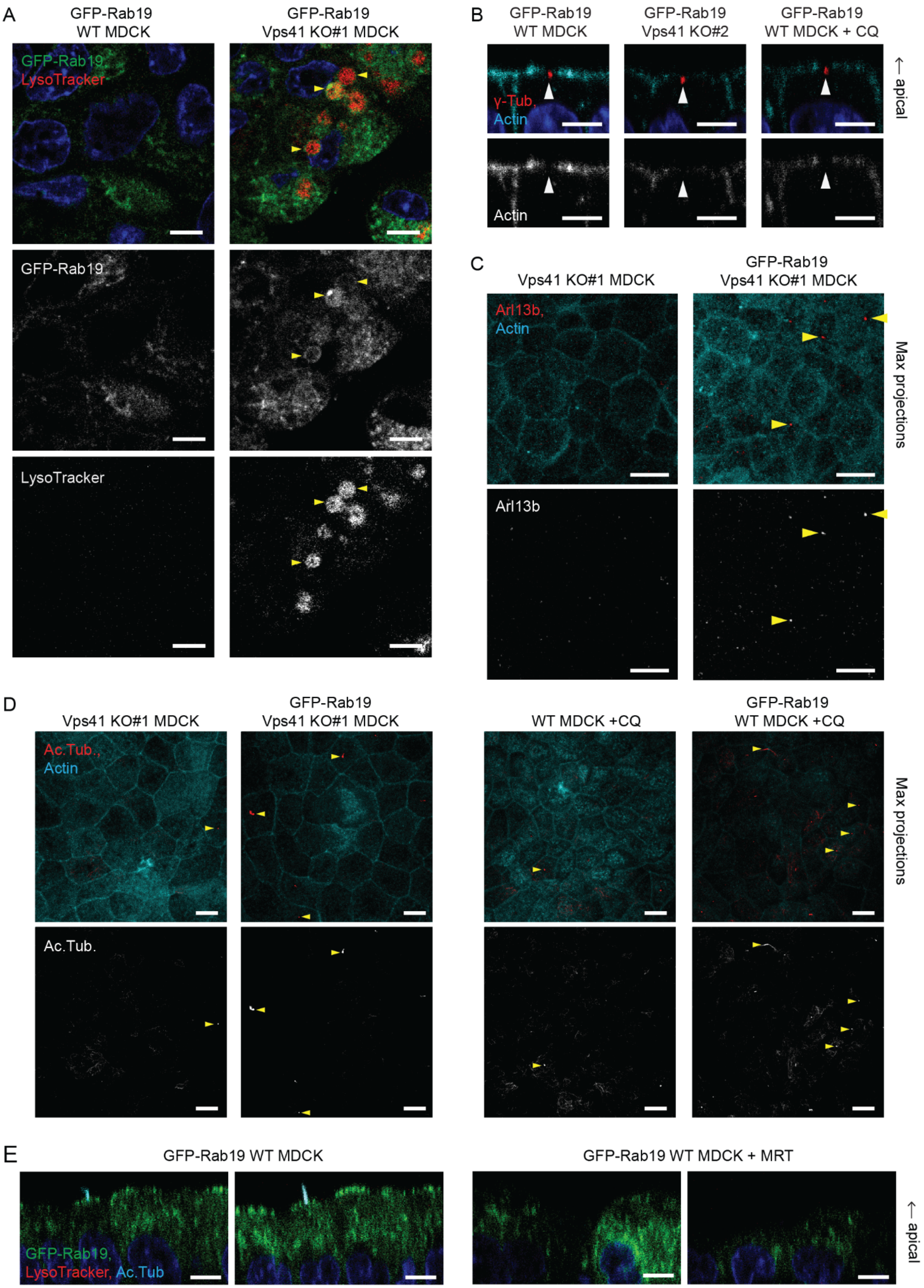

Journal of Cell Science • Supplementary information

Fig. S3. Additional data on Rab19 mislocalization in Vps41 KO, and rescue of actin-clearing and ciliation by Rab19 overexpression in Vps41 KO and CQ-treated MDCK.

A. MDCK cells expressing GFP-Rab19 on a Vps41 KO background, stained with LysoTracker. Scale bars 5 μm. Vps41 KO cells show increased localization of Rab19 to LE/L membranes (examples indicated with arrows).

B. GFP-Rab19-expressing WT, Vps41 KO and CQ-treated (10 μM for 3 days) MDCK cells, stained with γ-tubulin antibody and Phalloidin. Side views, scale bars 5 μm. Arrows: basal bodies. Rab19 overexpression rescued the clearing of cortical actin proximal to the basal body in Vps41 KO and CQ-treated cells (compare to Fig. 1C and Fig. S1A).

C. Vps41 KO MDCK cells with or without GFP-Rab19 overexpression, stained with Arl13b antibody and Phalloidin. MIPs, scale bars 10 μm. Arrows: examples of cilia. Rab19 overexpression rescued ciliation in Vps41 KO cells.

D. Vps41 KO and CQ-treated (10 μM for 3 days) MDCK cells with or without GFP-Rab19 overexpression, stained with acetylated α-tubulin antibody and Phalloidin. MIPs, scale bars 10 μm. Arrows: examples of cilia. Rab19 overexpression rescued ciliation in Vps41 KO and CQ-treated cells.

E. GFP-Rab19-expressing WT and MRT68921-treated (600 nM for 3 days) MDCK cells, stained with LysoTracker and acetylated α-tubulin antibody. Side views, scale bars 5 μm. Cells treated with the early-stage autophagy inhibitor MRT68921 showed a loss of.Rab19 localization to the apical cortex as well as a lack of primary ciliogenesis.

Journal of Cell Science • Supplementary information

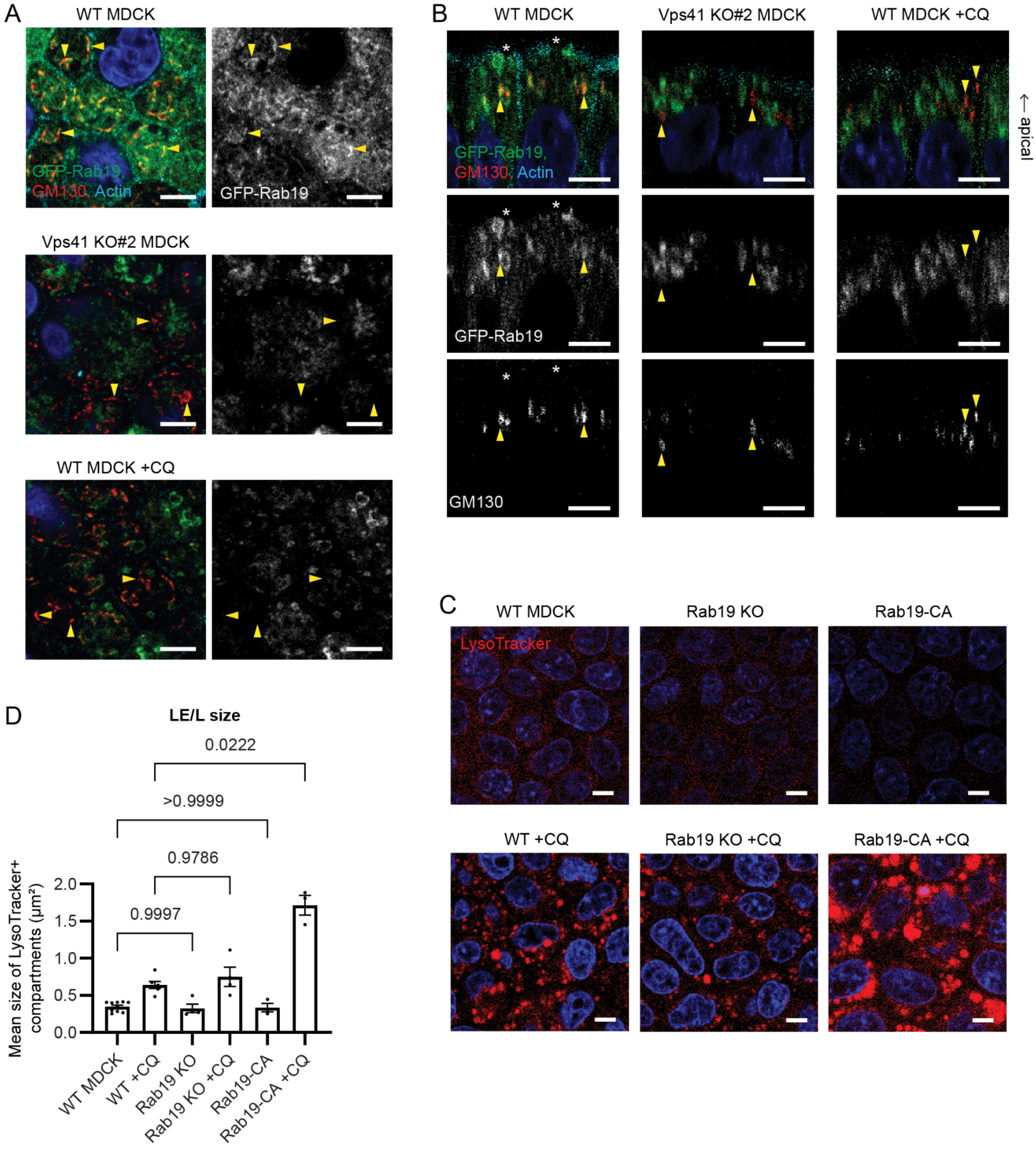

Fig. S4. Rab19 localizes to the Golgi and functions in trafficking to LEs.

A. MDCK cells expressing GFP-Rab19 on a WT background with or without CQ treatment, or GFP-Rab19 on a Vsp41 KO background, stained with GM130 (cis-Golgi marker) antibody, Phalloidin, and Hoechst. Arrows: examples of Golgi compartments. Scale bars 5 µm. Rab19 localizes to the Golgi in WT MDCK, and the Golgi localization of Rab19 is reduced in Vps41 KO and CQ-treated cells.

B. Side views from experiments as shown in (A). Scale bars 5 µm. Yellow arrows point to examples of Golgi

Journal of Cell Science • Supplementary information

compartments, and white asterisks point to examples of apical actin clearing. In polarized MDCK monolayers, the Golgi is found on the apical side of the nucleus, not in close proximity to the apically-docked basal body, so Golgi localization of Rab19 is separate from its localization to the site of ciliogenesis. Both Golgi localization and basal-body localization of Rab19 are reduced in Vps41 KO and CQ-treated cells.

C. WT, Rab19 KO, and Rab19-CA-expressing MDCK cells, with or without CQ treatment (10 μM for 2 days), stained with LysoTracker. Scale bars 5 μm. Rab19-CA expression enhanced the CQ-induced enlargement of LE/Ls.

D. Quantition of LE/L size for WT, Rab19 KO, and Rab19-CA-expressing MDCK cells in untreated and CQ-treated conditions, from experiments as shown in (C).

Journal of Cell Science • Supplementary information

A

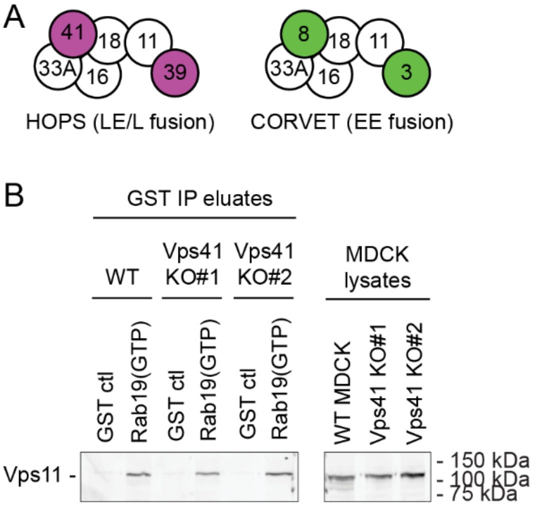

C

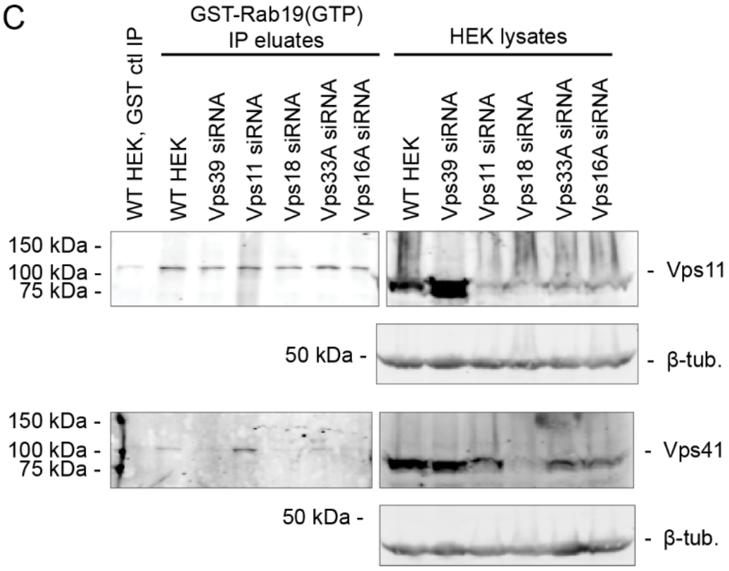

D

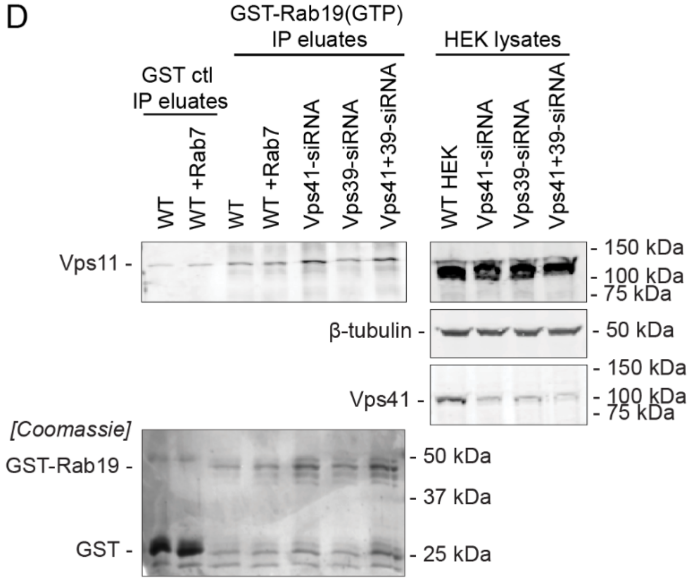

G

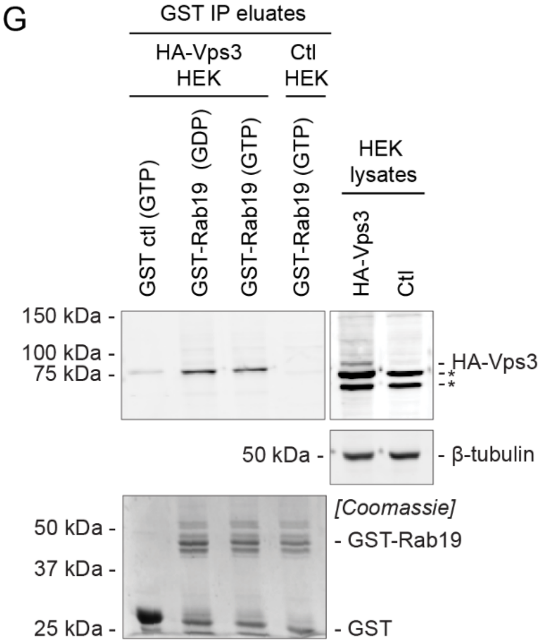

E

	Expression of target mRNA (% of WT):	
**siRNA target:**	*Fig. S5C*	*Fig. S5D*
Vps39 (alone)	47.2%	32.3%
Vps39 (w/ Vps41)		30.9%
Vps18	42.3%	
Vps33A	16.4%	
Vps16	7.7%	

H

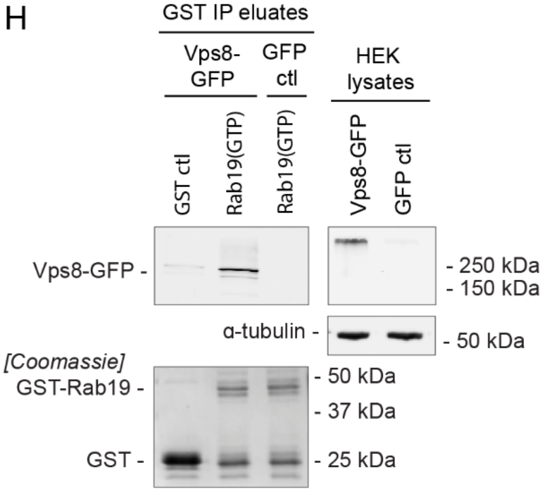

F

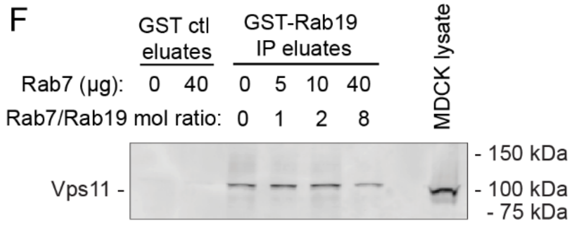

Fig. S5. Rab19 interacts with both HOPS and CORVET complexes.

A. Vps protein subunits of the HOPS and CORVET complexes. HOPS-specific subunits shown in purple, CORVET-specific subunits in green, and shared core subunits in white.
B. Anti-Vps11 western blot of GST-Rab19 immunoprecipitation (IP) assay on WT and Vps41 KO MDCK lysates, showing that Rab19 interacts with Vps11 regardless of presence of Vps41.
C. Anti-Vps11 and anti-Vps41 western blots of GST-Rab19 IP assay on WT and Vsp39-, Vps11-, Vps16-, Vps18-, and Vps33A-siRNA HEK293T lysates. Knockdown of any one of the core subunits severely disrupted Vps11 and Vps41 expression levels. Blot shown is representative of 3 biological replicates for WT vs. Vps39-siRNA conditions (besides the additional replicate of those conditions shown in (D)), and 2 biological replicates for all of the other siRNA conditions.
D. Anti-Vps11 and anti-Vps41 western blots of GST-Rab19 IP assay on WT and Vsp39-, Vps41-, and Vps39/41-siRNA HEK293T lysates, and on HEK293T lysates with addition of purified recombinant Rab7. Rab19-Vps11 interaction is not dependent on Vps39 or Vps41 and is not competitive with Rab7.
E. Validation of siRNA knockdowns of HOPS subunits in HEK293T cells. The mRNA expression levels of the listed targets in the respective siRNA-transfected HEK293T samples used for experiments as shown in (C) and (D) were assayed by RT-qPCR and normalized to GAPDH. (Vps11 and Vps41 expression were instead assessed by protein levels detected on the western blots).
F. Anti-Vps11 western blot of GST-Rab19 IP on WT MDCK lysates with increasing amounts of purified recombinant Rab7. The addition of Rab7 did not block Rab19 interaction with Vps11.
G. Anti-HA western blot of GST-Rab19 IP on HEK293T cells transfected with HA-Vps3 or untransfected control, showing that Rab19 interacts with CORVET subunit Vps3. Blot shown is representative of 3 biological replicates. Bands marked with (*) are nonspecific.
H. Anti-GFP western blot of GST-Rab19 IP on HEK293T cells transfected either with Vps8-GFP or with GFP control, showing that Rab19 interacts with CORVET subunit Vps8. Blot shown is representative of 3 biological replicates.

Journal of Cell Science • Supplementary information

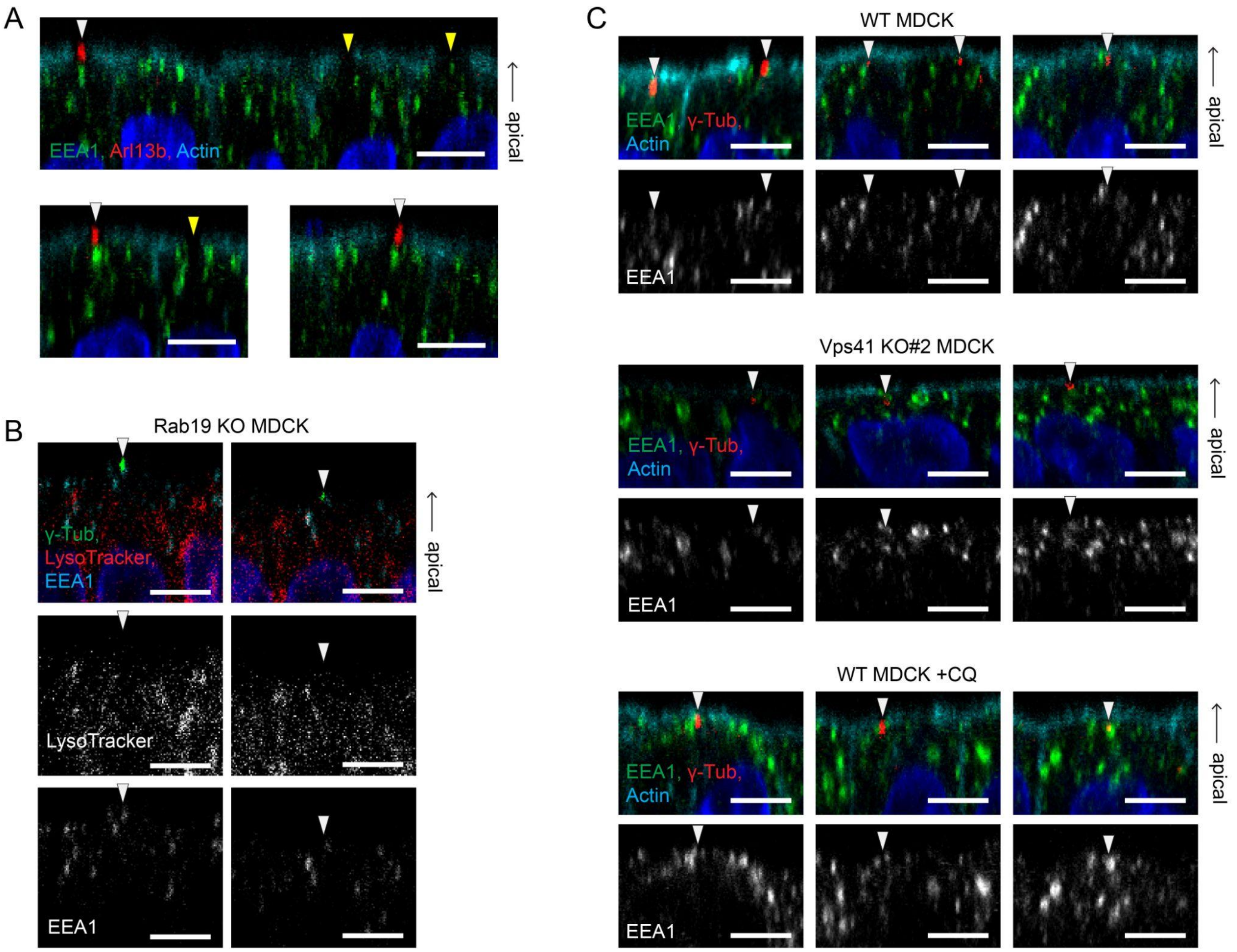

Fig. S6. EE targeting to basal body is not dependent on lysosomal fusion or Rab19. All scale bars in this figure 5 µm.

A. WT MDCK cells stained with Arl13b and EEA1 antibodies and Phalloidin, side views, showing EEA1 compartments at actin cortical clearing sites where cilia had not yet formed (yellow arrows) and at the base of cilia (white arrows).

B. Rab19 KO MDCK cells stained with γ-tubulin and EEA1 antibodies and LysoTracker, side views, showing EEA1 but not LysoTracker localized to the basal body.

C. WT, Vps41 KO, and CQ-treated MDCK cells, stained with γ-tubulin and EEA1 antibodies and Phalloidin, side views, showing EEA1 vesicles near the basal body.

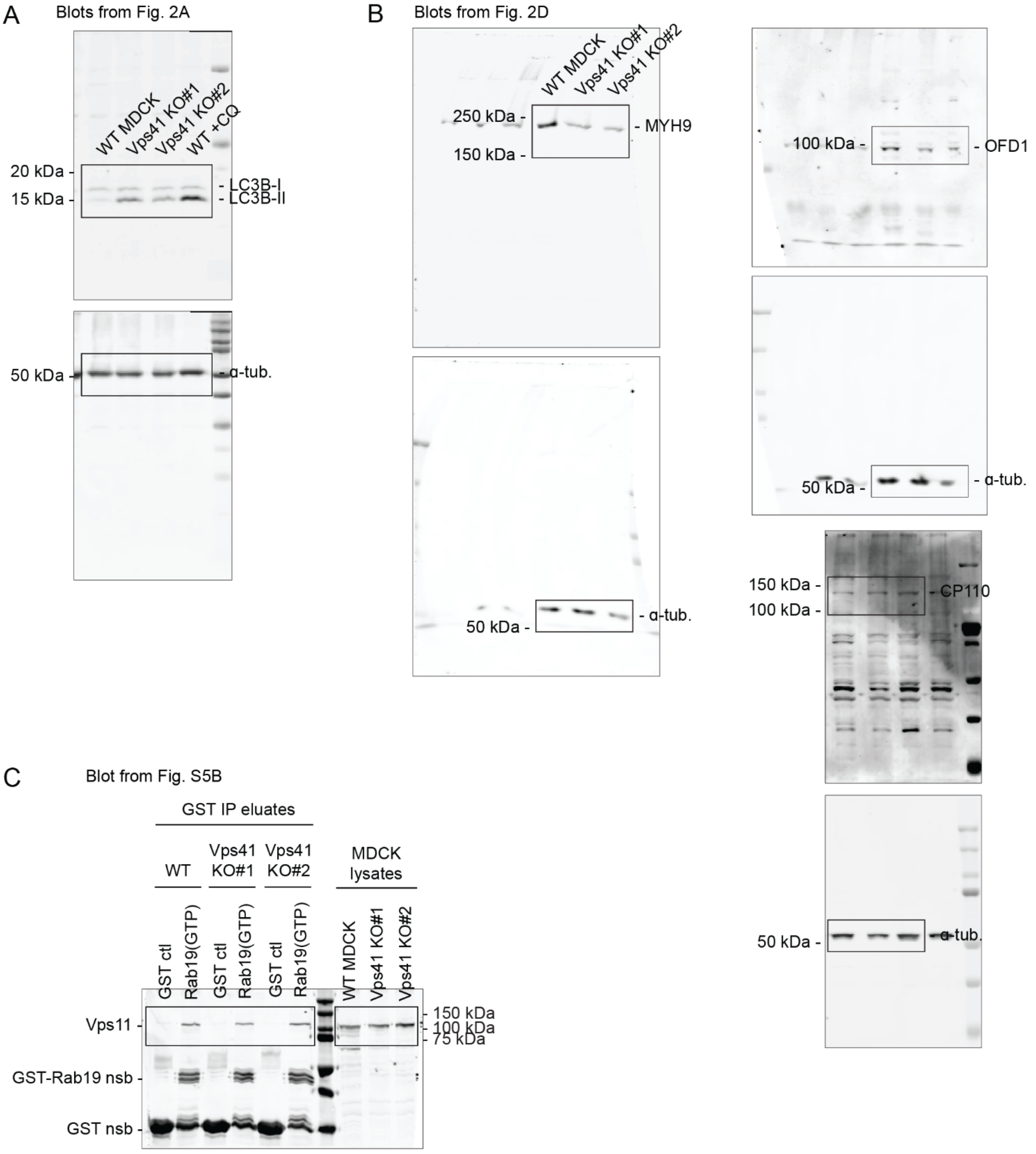

Journal of Cell Science • Supplementary information

D Blots from Fig. S5C

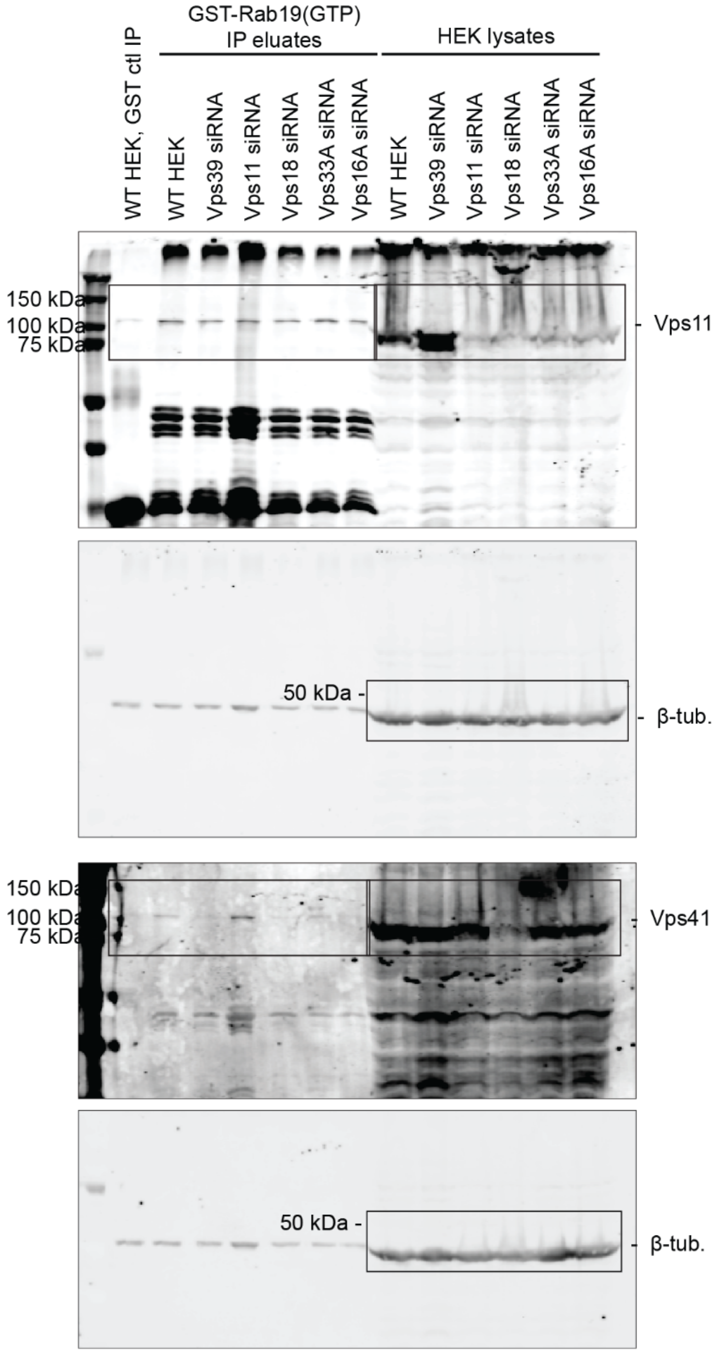

Journal of Cell Science • Supplementary information

E Blots from Fig. S5D

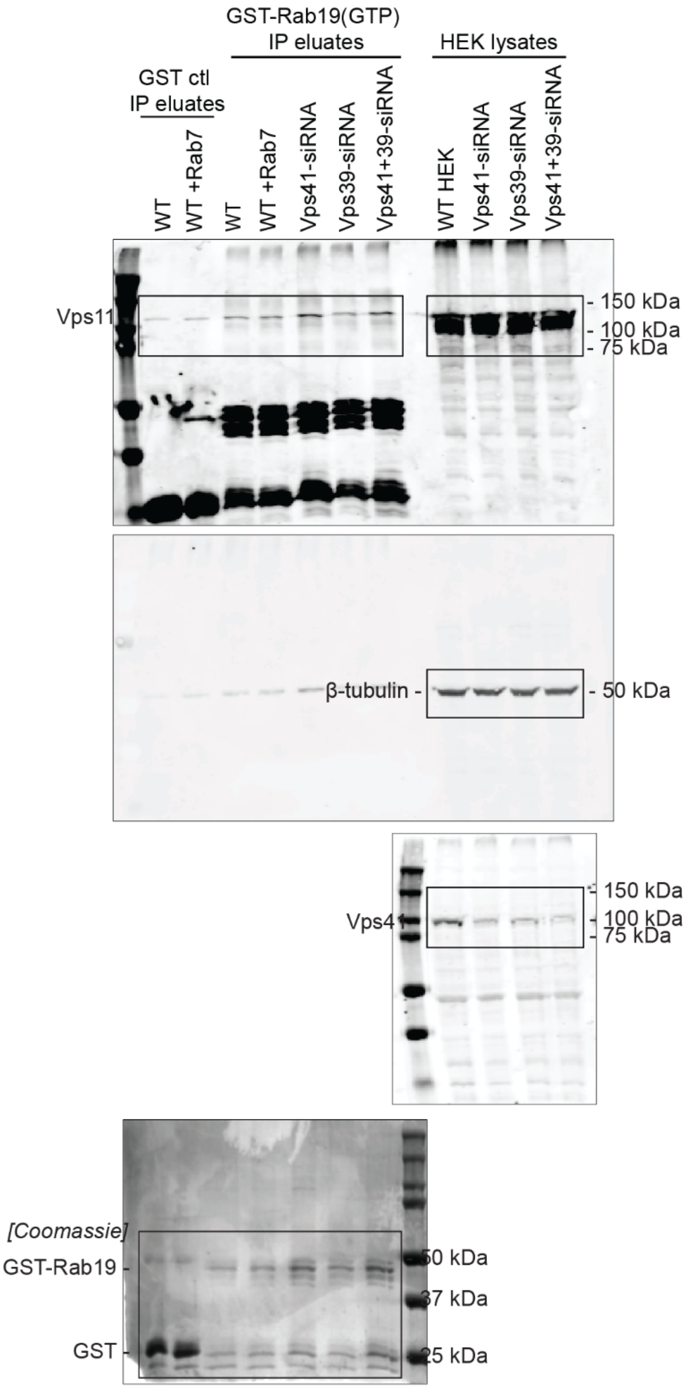

F Blot from Fig. S5F

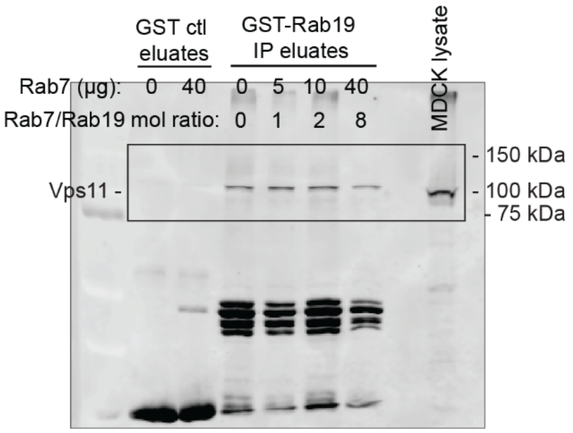

Journal of Cell Science • Supplementary information

G Blots from Fig. S5G

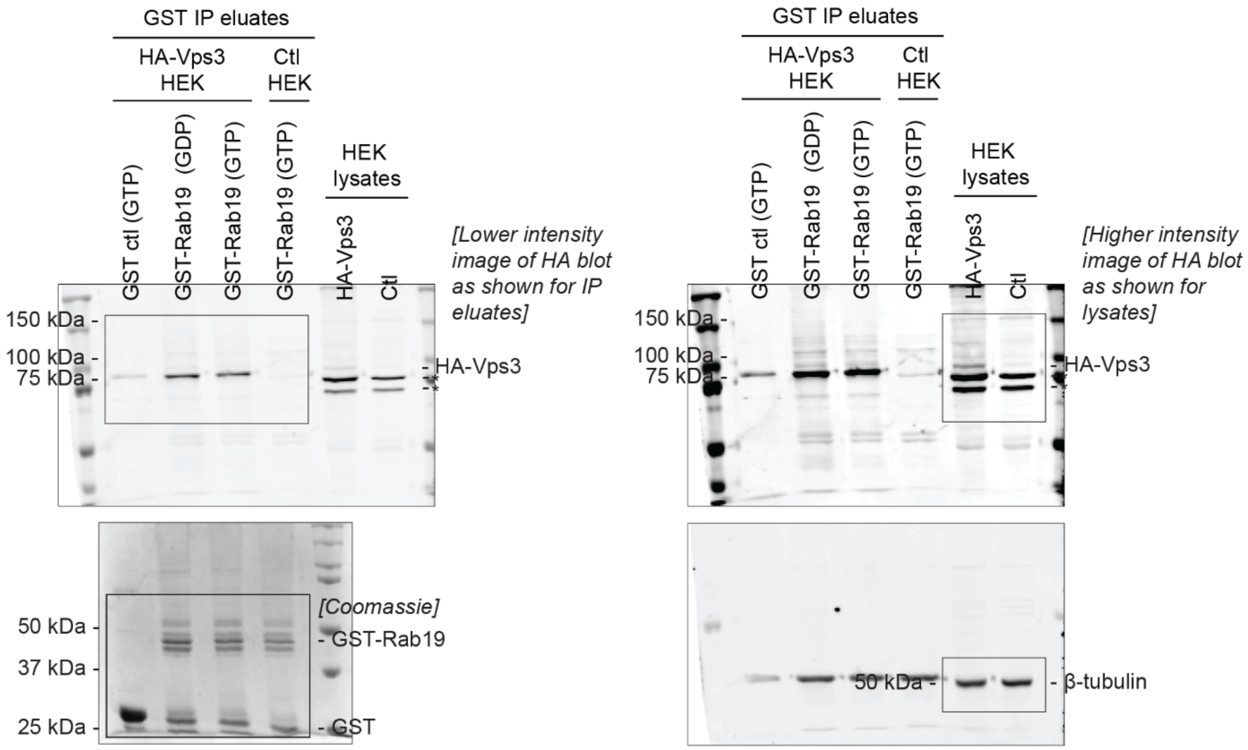

H Blots from Fig. S5H

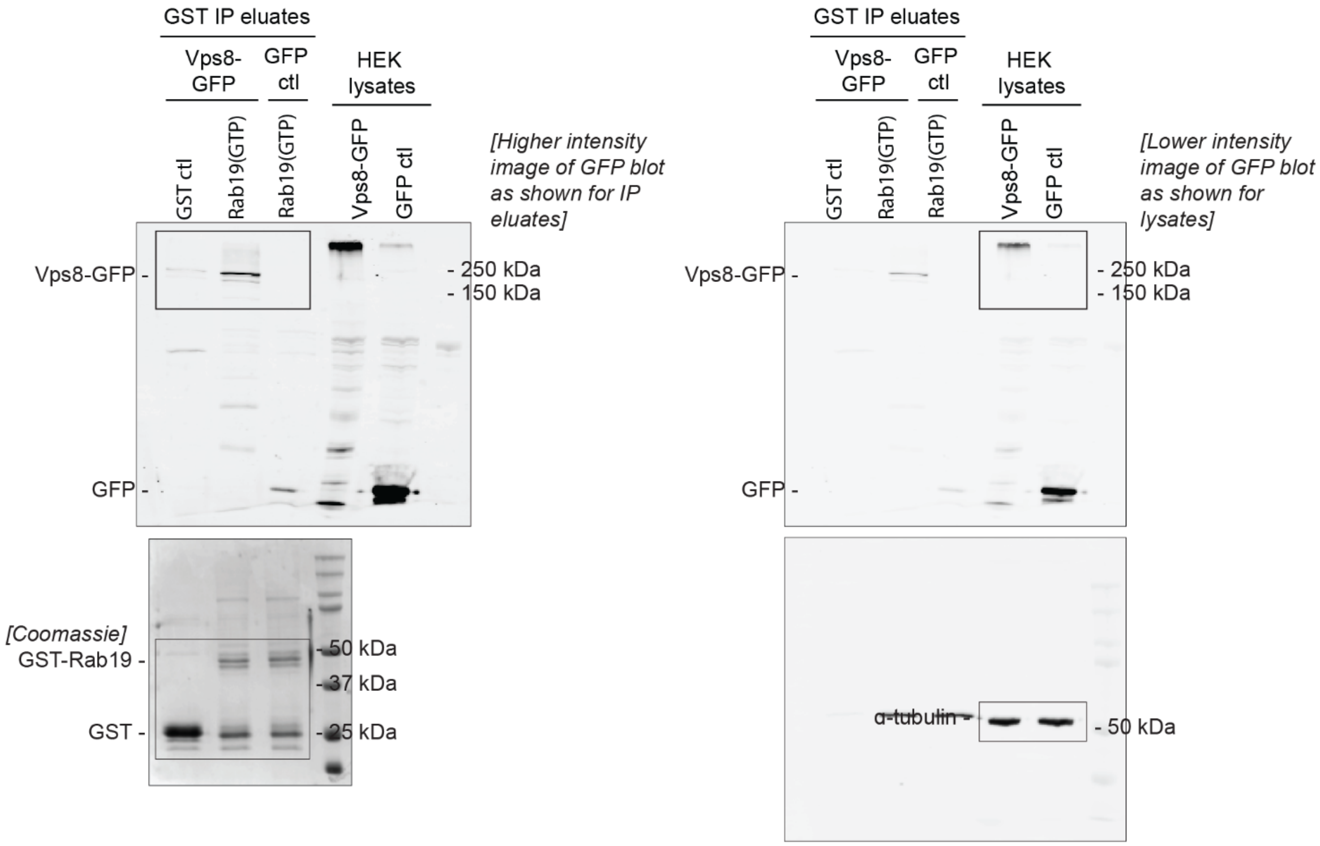

Journal of Cell Science • Supplementary information

Fig. S7. Blot transparency.

Full images of western blots from which cropped portions are shown in the following figure panels:

A. Blots from Fig. 2A.

B. Blots from Fig. 2D.

C. Blot from Fig. S5B.

D. Blots from Fig. S5C.

E. Blots from Fig. S5D.

F. Blots from Fig. S5F.

G. Blots from Fig. S5G.

H. Blots from Fig. S5H.

Journal of Cell Science • Supplementary information

Table S1. Cell lines used in this study.

**Cell line**	Source	Product number	Description
MDCK (Madin Darby Canine Kidney cells; MDCK.2)	ATCC (Manassas, VA, USA)	#CRL-2936	Renal polarized epithelial cell line, used for all experiments except where otherwise noted
mIMCD3 (mouse Inner Medullary Collecting Duct cells)	ATCC	#CRL-2123	Renal polarized epithelial cell line
RPE1 (human telomerase reverse transcriptase-immortalized Retinal Pigment Epithelial cells; hTERT-RPE1)	ATCC	#CRL-4000	Non-polarized cell line
HEK293T (Human Embryonic Kidney 293T cells)	ATCC	#CRL-3216	Highly transfectable cell line, used for lysates for the IP experiments requiring transfection with siRNAs, HA-Vps3, or Vps8-GFP

Journal of Cell Science • Supplementary information

Table S2. Antibodies and fluorescent dyes used for immunofluorescence and western blotting.

**Primary antibodies**	Source	Product number	Application (dilution)
γ-Tubulin	Sigma-Aldrich	#T5326	IF (1:500)
Acetyl-α-Tubulin	Cell Signaling Technology (Danvers, MA, USA)	#5335	IF (1:1000)
Arl13b	Antibodies Inc (Davis, CA, USA)	#73-287	IF (1:500)
LC3B	Novus Bio (Centennial, CO, USA)	#NB600-1384	WB (1:1000), IF (1:500)
MYH9	Sigma-Aldrich	#M8064	WB (1:2000)
OFD1	Abcam (Waltham, MA, USA)	#ab222837	WB (1:1000)
CP110	ProteinTech (Rosemont, IL, USA)	#12780-1-AP	WB (1:2000)
GM130	BD Biosciences (San Jose, CA, USA)	#610822	IF (1:250)
Vps11	Santa Cruz Biotechnology (Dallas, TX, USA)	#sc-515094	WB (1:200), IF (1:50)
Vps41	Santa Cruz Biotechnology	#sc-377271	WB (1:200)
β-tubulin	LI-COR (Lincoln, NE, USA)	#926-42211	WB (1:4000)
α-Tubulin	Sigma-Aldrich	#T6074	WB (1:5000)
α-Tubulin	Santa Cruz Biotechnology	#sc-23948	WB (1:200)
GFP	Thermo Fisher Scientific (Waltham, MA, USA)	#A11122	WB (1:2000)
HA	Santa Cruz Biotechnology	#sc-7392	WB (1:200)
EEA1	Thermo Fisher Scientific	#PA1-063A	IF (1:200)
**Secondary antibodies**	Source	Product number	Application
AF488 anti-mouse	Jackson ImmunoResearch (West Grove, PA, USA)	#715-545-150	IF (1:100)
AF594 anti-mouse	Jackson ImmunoResearch	#715-585-150	IF (1:100)
AF488 anti-rabbit	Jackson ImmunoResearch	#711-545-152	IF (1:100)
AF594 anti-rabbit	Jackson ImmunoResearch	#711-585-152	IF (1:100)
AF647 anti-rabbit	Thermo Fisher Scientific	#A-21245	IF (1:100)
AF488 anti-Mouse IgG2a	Thermo Fisher Scientific	#A-21131	IF (1:200)
AF568 anti-Mouse IgG1	Thermo Fisher Scientific	#A-21124	IF (1:200)
IRDye 680RD anti-mouse	LI-COR	#926-68072	WB (1:4000)

Journal of Cell Science • Supplementary information

IRDye 800CW anti-rabbit	LI-COR	#926-32213	WB (1:4000)
**Dyes**	Source	Product number	Application
LysoTracker Red DND-99	Thermo Fisher Scientific	#L7528	IF (1:2000)
Hoechst 33342	AnaSpec (Fremont, CA, USA)	#AS-83218	IF (1:1000)
AF568 Phalloidin	Thermo Fisher Scientific	#A12380	IF (1:40 from MeOH stock)
AF647 Phalloidin	Thermo Fisher Scientific	#A22287	IF (1:400 from DMSO stock)

Table S3. siRNAs and qPCR primers used for siRNA knockdowns of HOPS subunits.

**siRNAs (Qiagen)**	Product number
Hs_VPS39_1	#SI00110271
Hs_VPS11_6	#SI02778167
Hs_VPS18_3	#SI00760501
Hs_VPS33A_6	#SI03229436
Hs_VPS16_5	#SI03024483
Hs_VPS41_4	#SI00105665
**qPCR primers (IDT)**	Oligo sequence
qPCR-Vps39-F	AGCTGCCTCTGCAAATCGAC
qPCR-Vps39-R	ACATCTGCTGGCACAACGTC
qPCR-Vps18-F	GACTTCACCCCTTCCGAGC
qPCR-Vps18-R	TGCCCAAGTCAATGCGGAG
qPCR-Vps16-F	ACCCGAGAGCATCCAGTTTG
qPCR-Vps16-R	CCTCATGCAGGAACTCGTGG
qPCR-Vps33A-F	AAGGATCTGGGTGTCTTGGGA
qPCR-Vps33A-R	CCCTCCAGGTAGCACTCTTTGA
qPCR-GAPDH-F	TTAAAAGCAGCCCTGGTGAC
qPCR-GAPDH-R	CTCTGCTCCTCCTGTTCGAC
