# Supplementary material for: HOPS-dependent lysosomal fusion controls Rab19 availability for ciliogenesis in polarized epithelial cells
Source: J Cell Sci. 2023 Sep 4;137(5):jcs261047. doi: 10.1242/jcs.261047 (PMC10499034; doi:10.1242/jcs.261047)
Supplement: Supplementary file 1 [file joces-137-261047_review_history.pdf]

Journal of Cell Science

HOPS-dependent lysosomal fusion controls Rab19 availability for ciliogenesis in polarized epithelial cells

Huxley Kaminsky Hoffman and Rytis Prekeris
DOI: 10.1242/jcs.261047

Editor: David Bryant

Review timeline
Original submission: 8 February 2023
Editorial decision: 12 April 2023
First revision received: 20 June 2023
Accepted: 20 July 2023

Original submission

First decision letter

MS ID#: JOCES/2023/261047

MS TITLE: HOPS-dependent lysosomal fusion controls Rab19 availability for ciliogenesis in polarized epithelial cells

AUTHORS: Huxley Kaminsky Hoffman and Rytis Prekeris
ARTICLE TYPE: Research Article

We have now reached a decision on the above manuscript.

To see the reviewers' reports and a copy of this decision letter, please go to: https://submit-jcs.biologists.org and click on the 'Manuscripts with Decisions' queue in the Author Area. (Corresponding author only has access to reviews.)

As you will see, all three reviewers, who are experts in the field, gave quite favourable reports. However, the reviewers asked for a number of control experiments that will require amendments to your manuscript. I hope that you will be able to carry these experiments out in fairly short order because I would like to be able to accept your paper. I will need to send any revisions back to the reviewers.

In your revision, please provide a point-by-point response detailing how you have dealt with the points raised by the reviewers in the 'Response to Reviewers' box. Please attend to all of the reviewers' comments. If you do not agree with any of their criticisms or suggestions please explain clearly why this is so.

Please ensure that you clearly highlight all changes made in the revised manuscript. Please avoid using 'Tracked changes' in Word files as these are lost in PDF conversion.

Reviewer 1

Advance summary and potential significance to field

The manuscript by Hoffman and Prekeris describes that the role of the HOPS complex in ciliogenesis, previously described by the same group, is indirect since its depletion disrupts the

© 2023. Published by The Company of Biologists under the terms of the Creative Commons Attribution License (https://creativecommons.org/licenses/by/4.0/).

targeting of Rab19 to the basal body and consequently, actin clearing. This is in itself an important finding to report, even though the manuscript does not go far enough regarding the mechanism by which Rab19 regulates actin clearing. Nevertheless, the authors also show that Rab19 has a role in endolysosomal trafficking, that it interacts with a subunit of the v-ATPase and Vps8 subunit of the CORVET complex. Finally the authors found that CORVET-positive early endosomes are found at the basal body in contrast with late endosomes/lysosomes. Therefore, the manuscript deserves to be considered for publication in JCS. In general, this is a well designed and well performed study.

Comments for the author

Major issues:
1 - Two of the main issues of the manuscript, in its current form, are present in Fig. 1:
1.1 - In one experiment, the authors use KO#1 and #2, in another KO#1 and in the other KO#2. This also happens throughout the manuscript. The KO cell lines are clonal but they can differ. Therefore, both clones should be used, as in Fig. 1B.
1.2 - Chloroquine can have pleiotropic effects. For example, it is reported to also affect the Golgi and the endolysosomal system in general (e.g., see doi: 10.1080/15548627.2018.1474314). Therefore, results should be confirmed with an alternative compound such as bafilomycin A.
2 - For the experiments where the authors find a decrease in ciliation (Fig. 1G and 3F), an alternative ciliary marker (e.g., acetylated tubulin) must be used to exclude an effect on Arl13b recruitment or differences in ciliary membrane composition, as Arl13b is a ciliary membrane marker and acetylated tubulin stains the ciliary axoneme. Importantly, the % of ciliation is well below 10%, which is very low. Can the authors comment on the validity of the results with such low ciliation %?
3 - The study only uses GFP-tagged Rab19 and, at least by immunofluorescence, it is important to confirm the Rab19 localization using an antibody.
4 - In the paragraph starting in line 182, authors refer that GFP-Rab19 overexpression rescues actin clearing but the result is not shown in Fig. 3.
5 - Since the study reports a new interaction between Rab19 and Vps8, it would be important to confirm the interaction with the other CORVET subunit (Vps3),
but even more important is to determine if Vps8 is a Rab19 effector. This should be done by comparing the interaction between GTP and GDP conditions, as done for ATP6V1A (Fig. S1F).

Minor issues:
1 - Fig. 2B is related to Fig. 1E. Therefore, Fig. 2B should be moved to Fig. 1 or even added to the supplementary data since it is an additional control.
2 - The Discussion is already long, but it would be good to reinforce what the authors propose regarding the mechanism by which Rab19 could regulate actin clearing since the manuscript shows that HOPS is not involved, as suggested previously by the authors.

<u>Reviewer 2</u>

Advance summary and potential significance to field

Previous work from the Prekeris lab identified a requirement for Rab19 and the HOPS complex in clearing of cortical actin prior to ciliogenesis in MDCK cells

Given that HOPS displayed properties of a Rab19 effectors, it was postulated that Rab19 may function in actin clearing via HOPS. The current manuscript flips this hypothesis on its head and demonstrates that in the absence of HOPS, Rab19 becomes trapped on late endosomes and is no longer available to the basal body. Pharmacological inhibition of lysosomal fusion via chloroquine phenocopies loss of HOPS.

Quantitative analysis, and multiple experimental repeats contribute to a rigorous study. All cell biology is performed to high experimental standards. There is little to fault in the paper which frames the possible roles of lysosomal fusion in a new context.

© 2023. Published by The Company of Biologists under the terms of the Creative Commons Attribution License (https://creativecommons.org/licenses/by/4.0/).

Comments for the author

Minor points:

Duration of treatment with CQ needs to be indicated in corresponding figure legends.
Line 465 methods tells the rede that 'beginning approximately 0.5-2 hours after seeding the cells for the experiment, and refreshed daily.'

The biochemical experiments in Fig. 4C and 4D do not reach the same experimental standards. Repeating the immunoblots for these two figure panels should be considered.

The unsuccessful mapping of the Rab19 binding determinants inside the HOPS complex contributes little to the manuscript and Fig. 4B-D could be shifted to the supplement.

Fig. S1 provides an important qualification of the literature regarding role of autophagy in cilium assembly. In MDCK cells, no evidence that negative regulators of ciliogenesis are degraded via autophagy/lysosome. One minor suggestion would be to inhibit autophagy without inhibiting lysosomal degradation using a specific inhibitor (eg LY294002, MRT68921).

Line 246 'Rab19-HOPS interaction is independent of HOPS-specific subunits'. This statement is inaccurate. Better to write that the binding determinants for Rab19 are distributed across multiple HOPS subunits beyond Vps41 and Vps39.

Line 286-287: 'These observations support the idea that the interaction of CORVET with Rab19 might play a direct role in ciliogenesis'. The word 'support' seems too strong in for the sentence as the role of CORVET is not tested in the manuscript. Prefer 'is consistent with'

Reviewer 3

Advance summary and potential significance to field

Hoffman and Prekeris describe a role of Vps41 and HOPS in regulating ciliogenesis through an indirect mechanism involving effects on the availability of Rab19 in the apical cell periphery, where this lab showed previously a role of Rab19 in apical actin clearing needed for ciliogenesis. This is interesting because it opens the door to Rab19 having two distinct functions in ciliogenesis and endolysosomal function, and it suggests a mechanism by which the endolysosomal network may communicate to cilia.

Comments for the author

The study is largely descriptive in nature, and the data interpretation largely involves drawing inferences from immunocytochemical protein localization. However, the data that are presented appear to be of high quality and I think that the inferences drawn and interpretations proposed are reasonable. The finding that impairing lysosome function affects Rab19 availability at the apical cell cortex is, in my opinion, highly interesting in itself.

I do have one critical comment, or perhaps a suggestion is a better word... Can the authors say a little more about implications for ciliary defects in Joubert syndrome? My understanding is that INPP5E deficiency does not prevent ciliogenesis - I think Cilia form but have defects in composition and function. Can the authors be a little more explicit about their hypothesis re INPP5E mutations on cilia formation versus ciliary function?

© 2023. Published by The Company of Biologists under the terms of the Creative Commons Attribution License (https://creativecommons.org/licenses/by/4.0/).

First revision

Author response to reviewers' comments

We want to thank the reviewers for excellent suggestions. We have incorporated the vast majority of them in new edited manuscript. Additionally, we also added few experiments testing the role of Chloroquine in RPE1 cells (non-polarized epithelial cells that use intracellular ciliation pathway) and mIMCD3 (mice renal epithelial cells that use extracellular ciliation pathway). Interestingly, while Chloroquine inhibited ciliation in mIMCD3 cells, it had no effect on ciliation of RPE1 cells, suggesting that ciliation defects described in this manuscript only apply to polarized epithelial cells. All these new data was also added as new Figures. We believe that this new data, as well as experiments suggested by the reviewers, significantly enhanced the manuscript.

Reviewer 1

Major issues:
1) - Two of the main issues of the manuscript, in its current form, are present in Fig. 1:
1.1 - In one experiment, the authors use KO#1 and #2, in another KO#1 and in the other KO#2. This also happens throughout the manuscript. The KO cell lines are clonal but they can differ. Therefore, both clones should be used, as in Fig. 1B.

We have added data to illustrate the following phenotypes in the other Vps41 KO MDCK clone that was not previously shown:
- Actin cortical clearing is defective in Vps41 KO#2 (Supp. Fig. S1A).
- Ciliation is defective in Vps41 KO#1 (Supp. Fig. S1B).
- LC3B-II protein level is elevated in Vps41 KO#2 (Fig. 2A).
- MYH9, OFD1, and CP110 protein levels are not elevated in Vps41 KO#2 (Fig. 2D).
- Rab19 is aberrantly accumulated on LE/Ls in Vps41 KO#1 (Supp. Fig. S3A).
- Rab19 overexpression partially rescues ciliation in Vps41 KO#1 (Supp. Fig. S3C and D).

As a result, most of the analyses now use both KO clones.

1.2 - Chloroquine can have pleiotropic effects. For example, it is reported to also affect the Golgi and the endolysosomal system in general (e.g., see doi: 10.1080/15548627.2018.1474314). Therefore, results should be confirmed with an alternative compound such as bafilomycin A.

We attempted to test the effects of BafA on ciliation by performing similar experiments as with CQ, but we found that the MDCK cells were unable to withstand BafA treatment at an effective concentration for the 3-day duration used for our ciliation experiments. BafA concentrations of 5 nM or higher for 3 days resulted in cell death, while 2 nM or lower BafA showed little to no effect on lysosomes and therefore little to no effect on ciliation.
However, we found that treatment with the autophagy inhibitor MRT68921 produced similar effects as CQ in that Rab19 was depleted from the site of ciliogenesis and actin-clearing and ciliation were blocked. This is now shown in Fig. 2C and Supp. Fig. S3E.

2) - For the experiments where the authors find a decrease in ciliation (Fig. 1G and 3F), an alternative ciliary marker (e.g., acetylated tubulin) must be used to exclude an effect on Arl13b recruitment or differences in ciliary membrane composition, as Arl13b is a ciliary membrane marker and acetylated tubulin stains the ciliary axoneme. Importantly, the % of ciliation is well below 10%, which is very low. Can the authors comment on the validity of the results with such low ciliation %?

We have added data with staining for acetylated tubulin as an alternative ciliary marker to confirm the ciliation defects of Vps41 KO and CQ-treated MDCK cells (Supp. Fig. S1B), and the rescue of those ciliation defects by Rab19 overexpression (Supp. Fig. S3D). We have also added data showing the CQ-induced decrease in ciliation with acetylated tubulin staining in another polarized renal epithelial cell line, mIMCD3 cells (Fig. 4A,C).

As noted in the text describing the results in Fig. 1, the low % ciliation is attributable at least in part to the short time span of the ciliation experiments (3 days, vs. typically we would use 7 days), which was selected to minimize effects of the CQ treatment on cell viability. To ensure the validity

© 2023. Published by The Company of Biologists under the terms of the Creative Commons Attribution License (https://creativecommons.org/licenses/by/4.0/).

of the results in spite of the low % ciliation, we performed high numbers of biological replicates for these experiments (note that each data point on the graphs represents the mean of the technical replicates from a separate biological replicate) to show statistical significance.

3) - The study only uses GFP-tagged Rab19 and, at least by immunofluorescence, it is important to confirm the Rab19 localization using an antibody.

Unfortunately, there are no good anti-Rab19 antibodies that would work for IF. We have tried couple times to generate one in house, but the antibodies that we got only works (marginally) for western blotting.

4) - In the paragraph starting in line 182, authors refer that GFP-Rab19 overexpression rescues actin clearing, but the result is not shown in Fig. 3.

We have added data to illustrate the rescued actin-clearing in GFP-Rab19-overexpressing Vps41 KO and CQ-treated cells (Supp. Fig. S3B).

5) - Since the study reports a new interaction between Rab19 and Vps8, it would be important to confirm the interaction with the other CORVET subunit (Vps3), but even more important is to determine if Vps8 is a Rab19 effector. This should be done by comparing the interaction between GTP and GDP conditions, as done for ATP6V1A (Fig. S1F).

We have added data showing that Rab19 interacts with the other CORVET subunit, Vps3 (Supp. Fig. S5G).

In the new immunoprecipitation experiment testing Rab19 interaction with Vps3, we also tested GTP/GDP dependence, and found that the interaction could be detected in both GTP and GDP conditions (Supp. Fig. S5G). However, since the difference in binding affinities can be concentration-dependent, and since this experiment used overexpressed HA-Vps3 (due to the lack of a suitable antibody to detect endogenous Vps3) combined with purified GST-Rab19, we do not consider this result conclusive as to whether Rab19-CORVET interaction is GTP-dependent at the relevant physiological concentrations in cells.

Minor issues:
1) - Fig. 2B is related to Fig. 1E. Therefore, Fig. 2B should be moved to Fig. 1 or even added to the supplementary data since it is an additional control.

We have moved Fig. 2 (including Fig. 2B) entirely to the supplement, where it now appears as Supp. Fig. S2.

2) - The Discussion is already long, but it would be good to reinforce what the authors propose regarding the mechanism by which Rab19 could regulate actin clearing since the manuscript shows that HOPS is not involved, as suggested previously by the authors.

This study did not address the question of the mechanism by which Rab19 regulates actin cortical clearing, but the study that identified Rab19 as regulator of actin clearing for ciliogenesis (PMID: 33561422) showed that disrupting actomyosin contractility by treatment with a Rho-kinase (ROCK) inhibitor could rescue the actin-clearing defect of Rab19 KO MDCK cells, suggesting that Rab19 mediates actin clearing by antagonizing Rho/ROCK-dependent actomyosin contractility. We would speculate that Rab19 probably serves to recruit some actin-remodeling factor (e.g. some negative regulator of the Rho/ROCK axis) to the apically docked basal body. However, the identity of that factor remains to be determined. We have added to the text in the Conclusions section of the Discussion to clarify this interpretation.

Reviewer 2

Minor issues:
1) - Duration of treatment with CQ needs to be indicated in corresponding figure legends.
Line 465 methods tells the reader that 'beginning approximately 0.5-2 hours after seeding the cells for the experiment, and refreshed daily.'

© 2023. Published by The Company of Biologists under the terms of the Creative Commons Attribution License (https://creativecommons.org/licenses/by/4.0/).

We now specify the duration of the CQ treatment for each experiment in the figure legends.

2) - The biochemical experiments in Fig. 4C and 4D do not reach the same experimental standards. Repeating the immunoblots for these two figure panels should be considered.
The unsuccessful mapping of the Rab19 binding determinants inside the HOPS complex contributes little to the manuscript and Fig. 4B-D could be shifted to the supplement.

We opted to shift these Rab19 binding results to the supplement, where they now appear in Supp. Fig. S5.

3) - Fig. S1 provides an important qualification of the literature regarding role of autophagy in cilium assembly. In MDCK cells, no evidence that negative regulators of ciliogenesis are degraded via autophagy/lysosome. One minor suggestion would be to inhibit autophagy without inhibiting lysosomal degradation using a specific inhibitor (eg LY294002, MRT68921).

We thank the reviewer for suggesting this experiment, which yielded a very interesting result. When we tested the autophagy inhibitor MRT68921 in MDCK cells, we found that MRT68921 treatment produced similar defects in actin cortical clearing and primary ciliogenesis as seen in Vps41 KO and CQ-treated MDCK (Fig. 2C), and, importantly, Rab19 localization to the apical cortex and site of ciliogenesis was also lost in MRT68921-treated cells (Supp. Fig. S3E).

Thus, although we had supposed that this experiment would be a way to distinguish between the hypotheses of Rab19 mislocalization vs. disrupted autophagy being the mechanism for the actin-clearing and ciliogenesis defects of Vps41 KO and CQ-treated cells, it turned out that the result did not distinguish between those two hypotheses, since Rab19 was also mislocalized upon treatment with the specific autophagy inhibitor. The result is consistent with our model, since the actin-clearing and ciliogenesis defects of MRT68921-treated cells could be due to the lack of Rab19 at the site of ciliogenesis, but it would also be consistent with a model in which autophagic degradation of negative regulators is required to enable ciliogenesis.

However, this result is informative in that it provides an additional example of a condition in which defects in actin-clearing and ciliogenesis correlate with a lack of Rab19 enrichment at the site of ciliogenesis, bolstering the evidence for the role of Rab19 in this process. Furthermore, it raises the possibility that in some of the previous studies that concluded autophagy was required for ciliogenesis, Rab19 mislocalization could be part of the mechanism for the ciliogenesis defects seen in those autophagy-impaired conditions too.

4) - Line 246 'Rab19-HOPS interaction is independent of HOPS-specific subunits'. This statement is inaccurate. Better to write that the binding determinants for Rab19 are distributed across multiple HOPS subunits beyond Vps41 and Vps39.

We have changed the wording as suggested.

7) - Line 286-287: 'These observations support the idea that the interaction of CORVET with Rab19 might play a direct role in ciliogenesis'. The word 'support' seems too strong in for the sentence as the role of CORVET is not tested in the manuscript. Prefer 'is consistent with'

We have changed the wording as suggested.

Reviewer 3

1) - I do have one critical comment, or perhaps a suggestion is a better word... Can the authors say a little more about implications for ciliary defects in Joubert syndrome? My understanding is that INPP5E deficiency does not prevent ciliogenesis - I think Cilia form but have defects in composition and function. Can the authors be a little more explicit about their hypothesis re INPP5E mutations on cilia formation versus ciliary function?

© 2023. Published by The Company of Biologists under the terms of the Creative Commons Attribution License (https://creativecommons.org/licenses/by/4.0/).

We find somewhat conflicting literature on this question. The ciliary defects associated with INPP5E mutation or deficiency appear to vary with different experimental systems and tissue types. For example, decreased cilia formation was reported in polarized epithelial tissues in several in vivo models of INPP5E-related Joubert syndrome, including renal cysts from INPP5E-null mouse embryos (PMID: 19668215) and pronephric ducts and Kupffer's vesicles from INPP5E-morphant zebrafish larvae (PMID: 27401686, and PMID: 23022135).

On the other hand, in some other model systems, including mouse embryonic fibroblasts (PMID: 19668215), mouse neural stem cells (PMID: 26190144), mouse mesenchymal cells (PMID: 27998989), and INPP5E-related Joubert syndrome patient-derived fibroblasts (PMID: 29052317, PMID: 19668216), we see the reviewer's point that INPP5E deficiency has been found not to significantly reduce the number of cilia formed but rather to affect ciliary composition, length, stability, and/or signaling. It is also admittedly difficult to tell whether the reduced numbers of cilia which some of the other studies interpreted as indicating defects in cilia formation might instead be due to defects in cilia stability.

We have not found any literature directly assessing ciliogenesis in tissues of human patients with INPP5E-related Joubert syndrome, but overall these findings may suggest that INPP5E deficiency can prevent ciliogenesis in a cell-type- and context-dependent manner – perhaps particularly in polarized epithelial tissues – as well as perturbing other aspects of cilia function. That would be consistent with our model, since we now show that the impact of impaired lysosomal fusion on ciliogenesis is cell-type dependent.

We have revised the Discussion text to cite the literature that provides the strongest evidence for a role of INPP5E in ciliogenesis, and removed the assertion that INPP5E mutations "cause cilia defects in patients" since the literature we could find on this is in cell culture or animal models and does not really describe the ciliary phenotypes in human patients.

Second decision letter

MS ID#: JOCES/2023/261047

MS TITLE: HOPS-dependent lysosomal fusion controls Rab19 availability for ciliogenesis in polarized epithelial cells

AUTHORS: Huxley Kaminsky Hoffman and Rytis Prekeris
ARTICLE TYPE: Research Article

It is my absolute delight to tell you that your manuscript has been accepted for publication in Journal of Cell Science, pending standard ethics checks. This is a lovely manuscript, as all reviewers concurred (referee reportsappended below). Thanks again for choosing JCS as a home for your work.

Reviewer 1

Advance summary and potential significance to field

The authors provided substantial additional data to address the Reviewers' comments. Therefore, the manuscript is now suitable for publication in JCS.

Comments for the author

All the major and minor comments were properly addressed.

© 2023. Published by The Company of Biologists under the terms of the Creative Commons Attribution License (https://creativecommons.org/licenses/by/4.0/).

Reviewer 2

Advance summary and potential significance to field

The revision satisfies all my (minor) points.

Comments for the author

The effect of the ULK1/2 inhibitor on actin clearing is surprising but, as the authors correctly point out, in support of their model that Rab19-mediated clearing of the cortical actin is an essential step for apical ciliogenesis.

Reviewer 3

Advance summary and potential significance to field

This work elaborates an significant role of the HOPS complex and Rab19 in mediating ciliogenesis in polarized epithelial cells. This is an area of broad cell biological interest and is connected to many aspects of development and tissue physiology.

Comments for the author

The revised manuscript is improved from the initial submission, which was already very good. The authors have done a good job addressing my previous minor concern. I have no other critiques and complement the authors on an excellent study.

© 2023. Published by The Company of Biologists under the terms of the Creative Commons Attribution License (https://creativecommons.org/licenses/by/4.0/).
